# Rare CNVs and phenome-wide profiling highlight brain structural divergence and phenotypical convergence

**DOI:** 10.1038/s41562-023-01541-9

**Published:** 2023-03-02

**Authors:** Jakub Kopal, Kuldeep Kumar, Karin Saltoun, Claudia Modenato, Clara A. Moreau, Sandra Martin-Brevet, Guillaume Huguet, Martineau Jean-Louis, Charles-Olivier Martin, Zohra Saci, Nadine Younis, Petra Tamer, Elise Douard, Anne M. Maillard, Borja Rodriguez-Herreros, Aurélie Pain, Sonia Richetin, Leila Kushan, Ana I. Silva, Marianne B. M. van den Bree, David E. J. Linden, Michael J. Owen, Jeremy Hall, Sarah Lippé, Bogdan Draganski, Ida E. Sønderby, Ole A. Andreassen, David C. Glahn, Paul M. Thompson, Carrie E. Bearden, Sébastien Jacquemont, Danilo Bzdok

**Affiliations:** 1 Department of Biomedical Engineering, Faculty of Medicine, McGill University, Montreal, Quebec, Canada; 2 Mila - Quebec Artificial Intelligence Institute, Montréal, Quebec, Canada; 3 Centre de recherche CHU Sainte-Justine and University of Montréal, Montréal, Quebec, Canada; 4 LREN - Department of Clinical Neurosciences, Centre Hospitalier Universitaire Vaudois and University of Lausanne, Lausanne, Switzerland; 5 Human Genetics and Cognitive Functions, CNRS UMR 3571: Genes, Synapses and Cognition, Institut Pasteur, Paris, France; 6 Service des Troubles du Spectre de l’Autisme et apparentés, Centre Hospitalier Universitaire Vaudois and University of Lausanne, Lausanne, Switzerland; 7 Semel Institute for Neuroscience and Human Behavior, Departments of Psychiatry and Biobehavioral Sciences and Psychology, UCLA, Los Angeles, CA, USA; 8 School for Mental Health and Neuroscience, Maastricht University, Maastricht, the Netherlands; 9 MRC Centre for Neuropsychiatric Genetics and Genomics, Cardiff University, Cardiff, UK; 10 Division of Psychological Medicine and Clinical Neurosciences, School of Medicine, Cardiff University, Cardiff, UK; 11 Neuroscience and Mental Health Research Institute, Cardiff University, Cardiff, UK; 12 Neurology Department, Max-Planck-Institute for Human Cognitive and Brain Sciences, Leipzig, Germany; 13 NORMENT, Division of Mental Health and Addiction, Oslo University Hospital and University of Oslo, Oslo, Norway; 14 Department of Medical Genetics, Oslo University Hospital, Oslo, Norway; 15 KG Jebsen Centre for Neurodevelopmental Disorders, University of Oslo, Oslo, Norway; 16 Department of Psychiatry, Boston Children’s Hospital and Harvard Medical School, Boston, MA, USA; 17 Imaging Genetics Center, Stevens Neuroimaging and Informatics Institute, Keck School of Medicine of USC, Los Angeles, CA, USA; 18 TheNeuro - Montreal Neurological Institute (MNI), McConnell Brain Imaging Centre, Faculty of Medicine, McGill University, Montreal, Quebec, Canada

## Abstract

Copy number variations (CNVs) are rare genomic deletions and duplications that can affect brain and behaviour. Previous reports of CNV pleiotropy imply that they converge on shared mechanisms at some level of pathway cascades, from genes to large-scale neural circuits to the phenome. However, existing studies have primarily examined single CNV loci in small clinical cohorts. It remains unknown, for example, how distinct CNVs escalate vulnerability for the same developmental and psychiatric disorders. Here we quantitatively dissect the associations between brain organization and behavioural differentiation across 8 key CNVs. In 534 CNV carriers, we explored CNV-specific brain morphology patterns. CNVs were characteristic of disparate morphological changes involving multiple large-scale networks. We extensively annotated these CNV-associated patterns with ~1,000 lifestyle indicators through the UK Biobank resource. The resulting phenotypic profiles largely overlap and have body-wide implications, including the cardiovascular, endocrine, skeletal and nervous systems. Our population-level investigation established brain structural divergences and phenotypical convergences of CNVs, with direct relevance to major brain disorders.

A chief goal of modern neuroscience is understanding how genetic variation impacts brain organization and inter-individual differences in behaviour. Advances in genomic microarray technology streamlined the detection of copy number variations (CNVs)–deletions or duplications of chromosomal segments of >1,000 base pairs^1,2^.

This class of genetic mutations opens a unique window into the investigation of how neurogenetic determinants shape human behaviour, cognition and development^3,4^. Pathogenic CNVs that reoccur across individuals provide opportunities to study groups of individuals who carry the same deletion or duplication of a well-defined set of genes^5^. Moreover, CNVs have larger effects on phenotype than the low effect-size single-nucleotide polymorphisms often identified by genome-wide association studies^6^. Concretely, CNVs overall have been shown to detrimentally affect cognition and raise the risk for psychiatric conditions^4,7^. Nevertheless, it remains unexplained why many different CNVs escalate vulnerability for the same developmental and psychiatric disorders^4,8,9^.

The vast majority of large recurrent CNVs have been linked to more than one clinical diagnosis, including intellectual disability, autism spectrum disorders (ASD) and schizophrenia (SZ)^10–12^. These findings make a case that circumscribed genetic changes are rarely exclusively associated with a single clinical diagnosis^13^. Further, CNVs have demonstrable consequences even in seemingly unaffected middle and old-age carriers who show no overt signs of early-onset neuropsychiatric disorders. Recent evidence points to a broader spectrum of impacts from CNV status, ranging from physical traits to diabetes, hypertension, obesity, renal dysfunction^3,14,15^, as well as psychopathology^16^. Understudied body-wide CNV effects may contribute to the links of SZ-associated CNVs with diminished academic qualifications, occupation or household income^17^. In summary, this class of genetic variants affecting distant parts of the genome can be associated with various behavioural and clinical phenotypes^18,19^.

Despite many advances in genomic profiling, investigations into the corresponding brain signatures have only been performed for a few CNVs and mostly focused on a single variant at a time^20,21^. These parallel approaches to catalogue CNVs highlighted a wide spectrum of robust effects on brain structure^22,23^. Although distinct rare CNVs are associated with a range of brain alterations, they have been suggested to lead to a degree of similarity in associated behavioural phenotypes^9,24^. However, it remains unknown how similar CNVs are in terms of their effects on the brain and the phenome. Since deleterious CNVs are rare, such as 1 in 3,000 for 22q11.2 deletion^25^, previous investigations suffered from small samples of participants and a lack of phenotypic depth. Therefore, previous studies were chronically underpowered to paint a complete picture of CNVs in medicine. There is a need for a systematic investigation of intermediate brain measures and their phenotypic associations across several CNVs by means of a large well-phenotyped patient pool. The recent advent of population cohorts with rich phenotypic assessment batteries^26,27^ represents an untapped opportunity to conjointly examine a set of CNVs and characterize them at an unprecedented scale.

In the present study, we interrogated the largest existing biomedical data resource, the UK Biobank^28^, which allowed a head-to-head comparison of an envelope of CNVs. As a first step, we leveraged tools from machine learning, including linear discriminant analysis (LDA), to isolate CNV-specific brain morphology signatures from a multisite clinical cohort. These individuals carried 1 of 8 recurrent CNVs that are among the most widely studied CNV loci so far^11,23,29^. Deletions and duplications at loci 1q21.1, 15q11.2, 16p11.2 and 22q11.2 strike a balance between being frequent and having a significant impact on brain and behaviour^23^. Subsequently, the advantageous properties of LDA allowed us to carry over the CNV-specific whole-brain signatures from the clinical cohort to the large-scale UK Biobank cohort. The UK Biobank is ideally suited to tease apart the commonalities in phenotypic indicators across CNV alterations due to the breadth of available phenotypic annotations. We directly linked a rich portfolio of phenotypes to 8 CNV brain signatures in ~40,000 UK Biobank participants. Specifically, we performed separate phenome-wide association studies (PheWAS) for the 8 CNV-brain-imaging signatures across 977 phenotypes from 11 categories. In this way, we provide a population-level characterization of what unites and divides the 8 CNVs by detailing convergences and divergences from genomic variants to brain morphology to phenome. In an attempt to establish cornerstone evidence for the community, such a study can illuminate fundamental links between genetic variation and brain organization, with still unknown consequences to bodily systems.

## Results

### Dissecting different CNV effects on whole-brain morphology

We systematically analysed volumetric measures derived from brain-imaging scans in the clinical cohort comprising 846 total participants: 534 carried 1 of 8 recurrent CNVs (deletions and duplications of 1q21.1 distal, 15q11.2 BP1-BP2,16p11.2 proximal or 22q11.2 proximal), while 312 controls did not carry a CNV (Table 1). We parsed volume measures from these structural brain scans using a 400-region anatomical definition (Schaefer-Yeo reference atlas; see Online methods). To account for variation outside of our current primary scientific interest, each brain region volume was adjusted for intracranial volume, age, age^2^, sex and acquisition site for all downstream analysis steps. A schematic flow of all analysis steps is depicted in Supplementary Fig. 1.

**Fig. 1:**
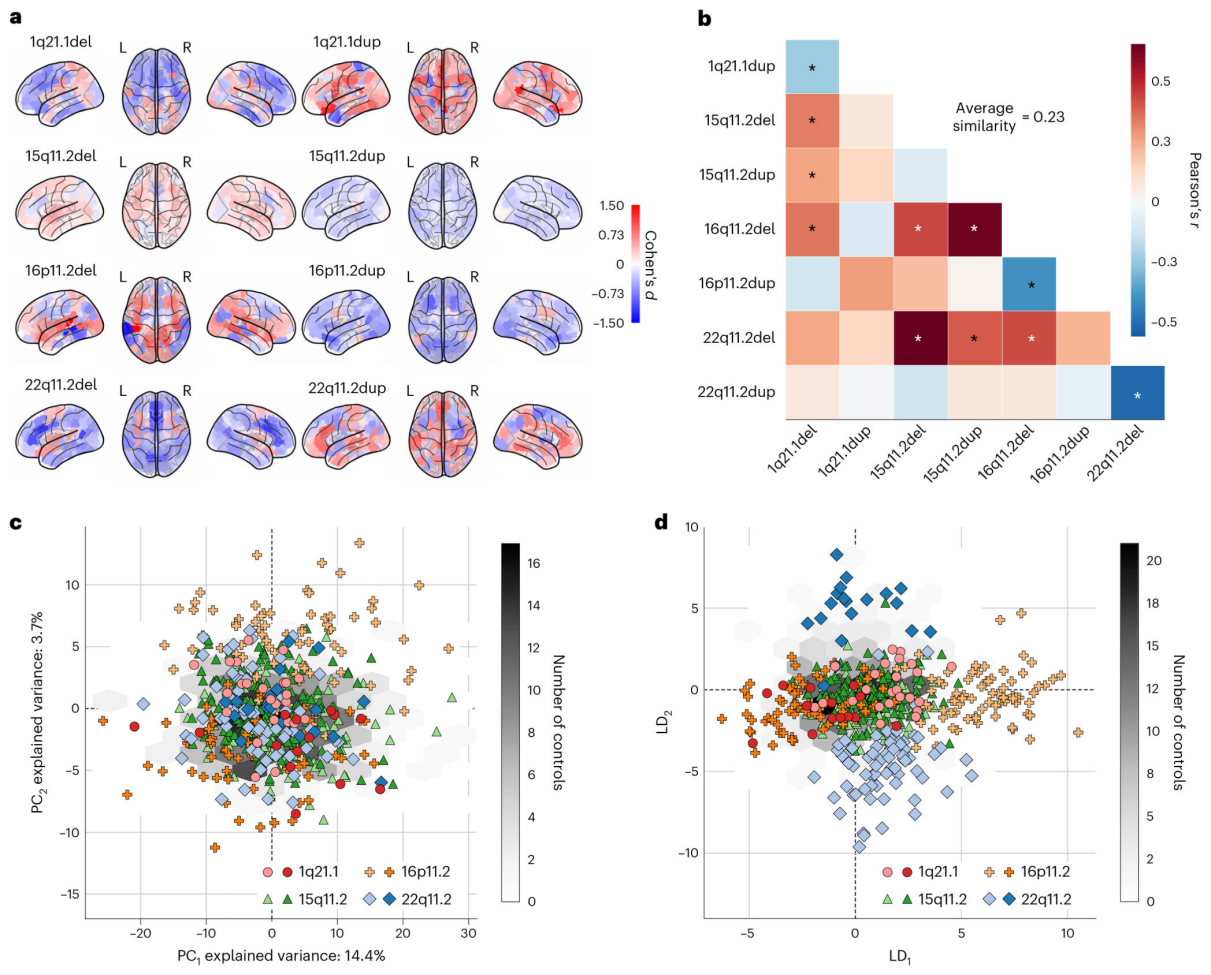
Eight CNVs lead to largely distinct spatial patterns of abnormalities in brain morphology. We analysed grey matter region volumes in 534 participants carrying 1 of 8 CNVs, and 312 controls. Regional volumes were adjusted for intracranial volume, age, age^2^, sex and acquisition site. **a**, Cohen’s *d* brain map quantifies the magnitude of structural change for each CNV. We computed Cohen’s *d* between CNV carriers and controls separately for each of the 400 brain regions (Schaefer-Yeo reference atlas). Our analysis reveals increased (red) and decreased (blue) brain volumes depending on the variation type. The uncovered patterns of volumetric changes confirm established knowledge on the regional increase and decrease across CNV loci^22,23^. **b**, Examining associations between Cohen’s *d* brain maps rendered on brain surface from each pair of CNVs. The wide range and low magnitude of Pearson’s correlations show that CNVs have distinct effects on brain volumes (darker red = more similar, darker blue = more dissimilar). Average similarity stands for the mean absolute Pearson’s correlation across all CNVs. 22q11.2 and 16p11.2 deletions and duplications show strong mirroring (opposing) effects. Asterisk denotes FDR-corrected spin permutation *P* values. **c**, Projecting brain volumes onto two dominant dimensions of variation using PCA. Although the first two dominant PCA components explain 18% of the variance, they are unrelated to differences between CNVs. The light and dark symbols represent deletions and duplications, respectively. The grey hexagonal bin plot represents the frequency of controls. Controls were not used to calculate the PCA and were projected post hoc. **d**, Projections of brain volumes to two dimensions using LDA. The first LDA dimension (LD_1_) mainly captures differences between 16p11.2 deletions and duplications, while the second LDA dimension (LD_2_) mainly captures differences between 22q11.2 deletions and duplications. Symbols and hexagonal binning plots were constructed in the same way as for the PCA approach. CNVs lead to distinct changes often represented by a predominant increase or decrease in the grey matter cortex that could effectively be described using low-dimensional representations derived by LDA models.

As a first step, we compared the effects on brain region volume measures for the 8 CNVs. Specifically, after normalizing (*z*-scoring) brain volumes across groups, that is, across the respective CNV carriers and controls, we examined the extent of volumetric divergence between carriers of each single CNV and controls by computing Cohen’s *d* (giving an effect size for the group difference) for each individual brain region (Fig. 1a). In doing so, for each examined CNV, we obtained a brain map of Cohen’s *d* effect sizes that summarizes magnitudes of CNV-induced structural abnormalities across the brain’s grey matter. We noted widespread smaller volumes in the majority of the examined atlas regions for the 1q21.1 deletion, 15q11.2 duplication, 16p11.2 duplication and 22q11.2 deletion. Conversely, a preferential increase in most regional volumes became apparent for the 1q21.1 duplication, 15q11.2 deletion, 16p11.2 deletion and 22q11.2 duplication. These findings align with well-known regional alterations identified in cohorts with patients carrying neurodevelopmental disorders^22^.

Each target CNV locus was characterized by an overall constellation of grey matter changes–a brain-wide CNV map of how particular CNV carriership results in systematic brain deviations from controls. To delineate the similarity among effect-size brain maps, we computed Pearson’s correlations between all 400 regional Cohen’s *d* values corresponding to each pair of CNVs (Fig. 1b). Statistical significance was assessed using a spin permutation test across the whole brain surface. We found a large disparity between Cohen’s *d* maps evidenced by the wide spectrum of Pearson’s correlations ranging from −0.51 to 0.63. We noted certain similarities, such as for deletions of 22q11.2 and 15qll.2 (*r* = 0.63, *P*_FDR-adj_ = 0 .03). Further, we observed a strong mirroring effect with significant anti-correlations between deletions and duplications of the same locus. Mirroring effects were strongest for 22q11.2 (*r* = −0.51, *P*_FDR-adj_ = 0.03), followed by 16p11.2 (*r*= −0.39, *P*_FDR-adj_ = 0.03). The average volumetric similarity measured by the average absolute Pearson’s correlations was *r* =0.23. Taken together, this cursory analysis indicated that spatial distributions of mutation-induced changes in brain morphology differed considerably across CNVs.

### Visualizing CNV differences in low-dimensional signatures

A drawback of the approach based on Cohen’s *d* lies in its univariate character, which considers each region separately, ignoring the respective remaining atlas regions. Hence, we next used dimensionality reduction techniques to obtain holistic summaries of the CNV carriers’ morphological profiles. We set out from the possibility that CNVs cause coordinated volume changes distributed across the entire brain. Therefore, we expected that an intrinsically brain-spanning pattern could be extracted that faithfully captures the induced morphological differences. Principal component analysis (PCA) is the most commonly used multivariate tool that is demonstrably most effective at representing linear latent factors. PCA can be interpreted as computing a new coordinate system such that the axes are oriented in the directions of the largest variation across the 400-region volume measures. We thus used PCA to project all CNV carriers’ regional volumes onto the two dominant directions of coherent whole-cortex variation (Fig. 1c). In the ensuing two-dimensional participant embedding, CNV carriers were scattered randomly without an apparent systematic relationship with each other. In other words, the results suggested that CNVs were not the primary source of the inter-individual variation in whole-cortex morphology in our cohort. Hence, a method without access to CNV-carriership status, such as PCA, could not provide a satisfying overall description of what drives structural brain deviations induced by specific CNVs.

Consequently, we turned to LDA as a pattern classification algorithm that is naturally capable of recovering a low-dimensional representation explicitly aimed at maximizing the separation among the 8 CNVs on the basis of the individuals’ brain morphometry measures. We re-expressed the brain-wide regional volumes as the two primary dimensions of structural variation under the LDA model (Fig. 1d). In particular, the leading dimension of the LDA-derived participant embedding captured the differences between 16p11.2 deletions and duplications. The second most explanatory dimension of the LDA-derived embedding mainly captured the differences between 22q11.2 deletions and duplications. This distribution of a single CNV locus along a single dimension again points at similar structural effects with opposite directions. In summary, LDA formed a new low-dimensional space in which the brain morphology of CNV carriers could be effectively identified, quantified and subsequently examined in further detail.

### Deriving CNV-specific intermediate phenotypes

**Fig. 2:**
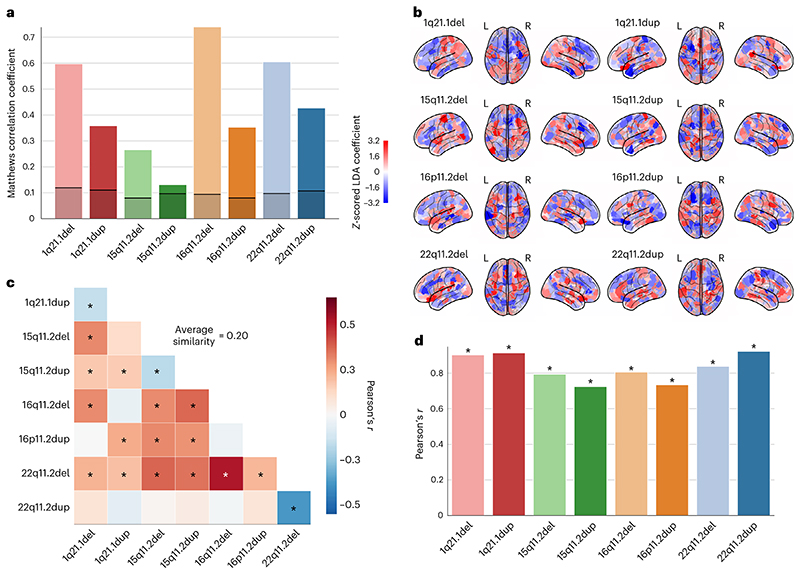
Pattern-learning models extract distinct intermediate brain phenotypes from CNV status. We estimated 8 LDA models to classify between controls and each of the 8 different CNVs. **a**, Classification performance of 8 distinct LDA models when telling apart controls and CNV carriers, given as Matthews correlation coefficient. All 8 CNVs are successfully classified on the basis of brain structure at above-chance accuracy as their performance exceeds that of an empirical null model (black line depicts upper 2.5 percentile threshold of the null distribution obtained by label shuffling). **b**, Prediction rule derived for each of the 8 CNV-specific LDA models projected on the brain (red/blue = positive/negative weight). The prediction rule is a CNV-specific brain signature and can be treated as an intermediate phenotype. **c**, Similarity between CNV-specific intermediate phenotypes. The wide range and low magnitudes of resulting Pearson’s correlations reflect the disparity in the captured intermediate phenotypes. Average similarity represents the mean absolute correlation across all CNVs. Asterisk denotes FDR-corrected spin permutation *P* values. **d**, Relationship between Cohen’s *d* brain maps and intermediate phenotypes. On the basis of FDR-corrected Pearson’s correlations, all 8 intermediate phenotypes appear to largely follow the respective Cohen’s *d* brain maps. LDA models identified and quantified CNV-specific intermediate phenotypes that effectively captured distinct morphometric differences between CNV carriers and the general population. Asterisks denote FDR-corrected spin permutation *P* values.

To supplement the multi-CNV classification model which explored differences between CNVs (described above), our next analysis step was to extract robust whole-brain signatures specific for each CNV that we could then use to study unseen participants in any number of external cohorts. Therefore, we constructed 8 LDA models of order one dedicated to the 8 CNVs. Notably, there was a considerable imbalance between the number of controls and CNV carriers (from 2-fold for 15q11.2 duplication to 22-fold for 1q21.1 duplication). Moreover, the number of model parameters to be estimated (at least 400 parameters associated with the 400 atlas regions) was larger than the number of participants. To remedy the challenges of this data scenario, our analysis pipeline combined bagging and regularization to prevent overfitting of the model hyperparameters (see details in Online methods). We evaluated the model performance indexed by out-of-sample prediction in brain scans unseen by the model using the Matthews correlation coefficient. All CNVs were successfully classified with a consistent above-chance accuracy (Fig. 2a). Chance-level accuracy was defined as the performance of an empirical null model obtained by label shuffling. High classification performance provides empirical evidence that these CNVs are characteristic of robust volumetric signatures.

After extracting predictive principles of structural brain deviations by means of LDA, each model included a collection of 400 coefficients associated with the atlas regions (Fig. 2b). These coefficients encapsulated a multivariate prediction rule which maximized the difference between controls and CNV carriers. In other words, each CNV-specific LDA model encapsulated an intermediate phenotype–a brain-wide volumetric signature that characterizes each CNV. To quantify the similarity between the derived intermediate phenotype representations, we compared them using Pearson’s correlation coefficient. Again, we observed certain similarities across the 8 CNVs, as well as mirroring effects between reciprocal CNVs (Fig. 2c). However, the wide range and low strength (average similarity *r* = 0.2) of obtained CNV-CNV similarities indicated that LDA models reflected the sizable diverging effects of CNVs on brain morphometry. The identified intermediate phenotypes bore a degree of similarity to the Cohen’s *d* brain maps (Fig. 2d). The strong positive Pearson’s correlations between the intermediate phenotypes and Cohen’s *d* brain maps were significant for all CNVs. In other words, LDA-derived (brain-global) patterns capture certain volumetric effects highlighted by previous (region-local) Cohen’s *d* analysis. Along with the high prediction accuracy, a degree of similarity with estimated region-wise Cohen’s *d* maps is an important step towards characterizing derived signatures in another dataset.

**Fig. 3:**
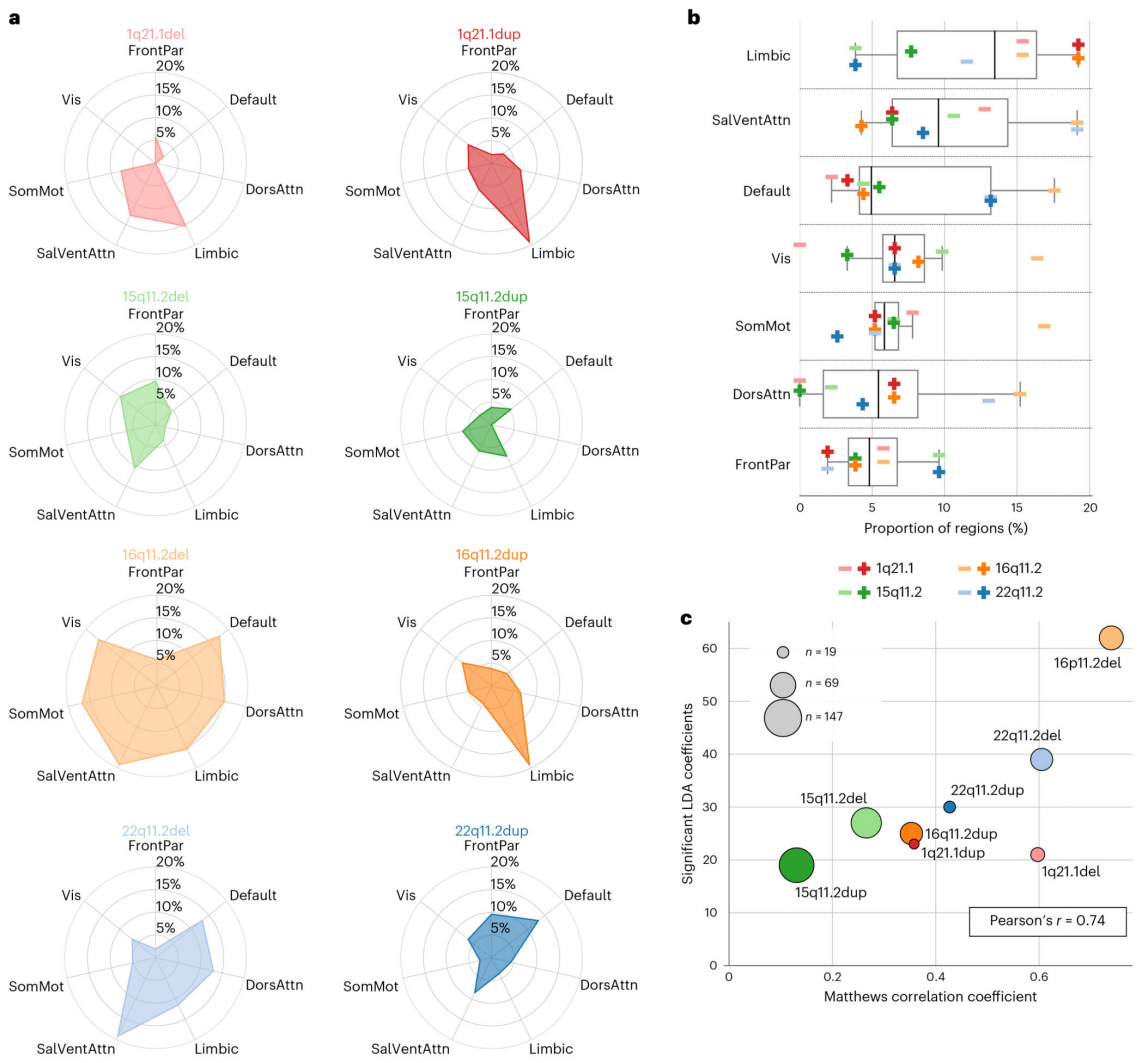
Intermediate brain phenotypes track structural changes with distinct impacts on large-scale networks. We identified which aspects of the LDA-derived prediction rule robustly contributed to classification success by calculating 100 bootstrapped LDA models for each CNV while sampling CNV carriers randomly. **a**, Percentage of statistically relevant LDA coefficients in a CNV carrier group among all the regions that belong to each brain network (one-sample bootstrap hypothesis test for non-zero mean with 10,000 replicates). For example, 16p11.2 deletion strongly affects most large-scale networks except the frontoparietal network. Altogether, the estimated LDA coefficients represent the backbone of each intermediate phenotype. Large-scale networks correspond to 7 Schaefer-Yeo networks; Vis, visual; FrontPar, frontoparietal; SomMot, somatomotor; DorsAttn, dorsal attention; SalVenAttn, salience ventral attention; Limbic; Default, default mode. **b**, Significant LDA coefficients grouped by the large-scale networks. The highest relative number of affected regions is in the limbic network. Conversely, regions in the frontoparietal network are targeted less frequently. **c**, Relationship between CNV effects and LDA performance. There is a significant positive correlation between the number of significant LDA coefficients and classifier performance, unlike for the sample size of the cohort (marker size). According to the 8 specific LDA models, CNVs affected predominantly high-level networks such as the limbic, salience and default-mode networks.

We further inspected the 400-region coefficients of each LDA model that captured the influence of each CNV on each brain region. By carrying out a one-sample bootstrap hypothesis test independently for each CNV, we assessed which region-specific model coefficients are robustly different from zero and, thus, robustly affected by CNVs. Specifically, during the learning of the coefficients of 1 of the 8 CNV-specific LDA models in 100 resampling iterations, we drew a different set of participants by drawing participants with replacement from the control participants and corresponding CNV carriers. Statistically relevant coefficients were robustly different from zero if their two-sided confidence interval (according to the 2.5/97.5% intervals of the bootstrap-derived distribution) did not include zero. Different CNVs affected (displayed statistically relevant coefficients) different cortical parcels that correspond to the 7 large-scale brain networks populating the cortex, as defined by our atlas (Fig. 3a). For example, while 16p11.2 duplication primarily affects 20% of all regions in the limbic network, 22q11.2 deletion affected 20% of regions in the salience ventral attentional network as well as more than 10% of regions in the limbic, dorsal attentional and default-mode networks. Across all examined CNVs and target brain networks, the 16p11.2 deletion affected the largest number of brain regions, while 15q11.2 duplication affected the lowest number of regions. Higher-order network circuits showed, on average, the relatively highest number of significant coefficients. Concretely, the limbic network had the highest relative number of affected regions, followed by the salience and default-mode networks (Fig. 3b). Together, the wide range of effects on the large-scale networks again highlights the diverging consequences of different CNVs on brain morphometry.

To further explore characteristic relationships among the 8 CNVs, we probed for a linear relationship of the number of salient LDA coefficients with LDA classifier performance and average brain-wide Cohen’s *d*. We found a significant positive Pearson’s correlation with classifier performance (*r* = 0.74, *P* = 0.04) (Fig. 3c) and mean absolute effect size (*r* = 0.75, *P* = 0.03). Furthermore, when we included sample size in the testing scheme, we found only a negative linear association with average Cohen’s *d* (*r* = −0.80, *P* =0.02), calling for careful interpretation of effect sizes, owing to the estimation of population mean in small samples. In sum, our collective findings highlighted how LDA models reflect CNV-specific changes in large-scale brain networks to form distinctive intermediate phenotypes.

### Lifting over phenotype patterns from the clinical cohort

**Fig. 4:**
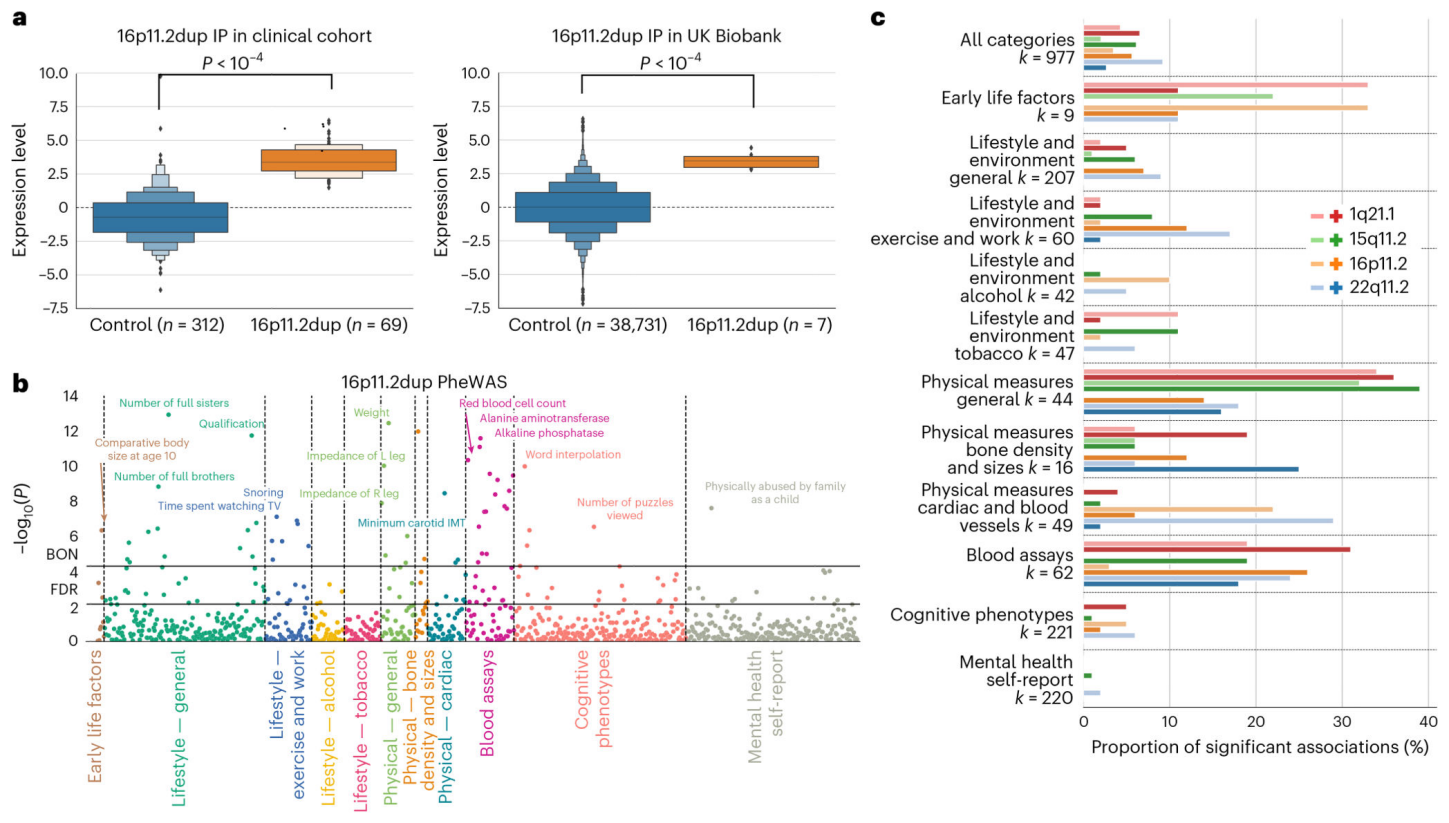
Using intermediate CNV phenotypes as a basis for phenome-wide association analysis. We performed a PheWAS by computing Pearson’s correlations between the expression of each of the 8 intermediate CNV phenotypes and 977 phenotypes spanning 11 categories in 39,085 UK Biobank participants. **a**, Letter-value (boxen) plot for the expression of 16p11.2 duplication intermediate phenotype is shown for the sake of illustration. The boxen plot depicts the distribution of quantiles for the expression scores computed by quantifying the presence of derived 16p11.2 duplication intermediate phenotype in both the clinical cohort (left) and the UK Biobank (right). On the basis of a two-sample bootstrap hypothesis test for difference of means with 10,000 bootstrap replicates, the 16p11.2 duplication carriers significantly differed in the expression level from controls both in the clinical cohort *(P* < 10^-4^) and the UK Biobank dataset (*P* < 10^-4^). **b**, PheWAS study using the CNV-specific intermediate phenotype. We calculated the Pearson’s correlations between the expression of 16p11.2 duplication intermediate phenotype and each of977 phenotypes. After the Bonferroni correction for multiple comparisons (BON), there were 55 significant associations, such as education score, haemoglobin concentration or physically abused by family as a child. There were 145 significant associations exceeding FDR correction. **c**, The relative number of significant correlations summarized for each of the 11 categories for each CNV in the UK Biobank. Most CNVs are strongly associated with multiple categories and their respective phenotypes. For example, up to 35% of phenotypes in general physical measures show a significant correlation with 4 CNV brain signatures. The light and dark symbols represent deletions and duplications, respectively. As an insight from the performed phenome-wide association analysis, CNV brain signatures are linked with multiple phenotypes across most categories but mainly in the general physical measures, blood assays and early life factors categories.

We built 8 separate LDA models that encapsulated CNV-specific intermediate phenotypes. By doing so, we could quantify the presence of each intermediate CNV phenotype for each participant. Hence, as an illustrative example, we compared the expression level of 16p11.2 duplication intermediate phenotype between the carriers of that CNV and controls. On the basis of a two-sample bootstrap hypothesis test for the difference of means with 10,000 bootstrap iterations (see Online methods), the resulting means of the intermediate phenotype expressions differed significantly between CNV carriers in the clinical sample and controls (*P* < 10^-4^) (Fig. 4a). As a critical step in our analysis, the CNV-specific volumetric signatures derived from our clinical population using LDA could be used in a phenotypically richer population data repository.

To carry over the intermediate phenotypes from the clinical cohort to the UK Biobank, we quantified the expression of each intermediate CNV phenotype for all 39,085 UK Biobank participants (Table 2). We first extracted brain volume measures from the 400 atlas regions, adjusting for several confound variables (see Online methods). We then calculated the participant-specific expression for all intermediate CNV phenotypes in the UK Biobank. It is important to stress that the intermediate phenotypes were derived in the clinical cohort. However, UK Biobank also contains several carriers of the analysed mutations. The generalizability of the derived intermediate phenotypes was indicated by the difference between the intermediate phenotype expression level of non-carriers and CNV carriers in the UK Biobank (for 16p11.2 duplication, *P* < 10^-4^ using a test identical to that above; Fig. 4a). Notably, we obtained similar results for all other 7 intermediate phenotypes (Supplementary Fig. 2). Carrying over CNV-associated magnetic resonance imaging (MRI) profiles computed in the clinical cohort to the UK Biobank was a critical step that allowed us to identify phenotype correlates of the CNV-associated MRI profiles in a population >500 times larger in participant number than our CNV cohorts.

### Charting phenome-wide associations of CNV signatures

The UK Biobank is the largest existing uniform brain-imaging dataset in terms of participant sample size and breadth of available phenotypic annotations. It provides 977 unique phenotypes spanning 11 different categories (Supplementary Fig. 3). We performed an exploratory PheWAS for the purpose of generating new candidate hypotheses. PheWAS allows investigation of the overall patterns of connections by charting associations among hundreds of non-imaging phenotypes and imaging-derived phenotypes. Specifically, we calculated Pearson’s correlations between the derived participant-specific expressions of the 8 intermediate CNV phenotypes and each of the 977 phenotypes provided by the UK Biobank resource (Fig. 4b). In our recurring example of the 16p11.2 duplication intermediate phenotype, 55 associations surpassed Bonferroni correction for multiple testing (including comparative body size at age 10, education score, haemoglobin concentration or physically abused by family as a child), while 145 associations surpassed false discovery rate (FDR) correction. In other words, individuals with greater similarity to the 16p11.2 duplication MRI profiles showed a stronger association with levels of education or blood assay biomarkers.

To gain additional insight, we summarized the phenotypic association profiles by domain. To this end, we calculated the relative number of association hits for each of the 11 phenotypic domains (using the more stringent Bonferroni correction) as a ratio between the number of significant associations and the number of phenotypes in each category. The highest relative number of associations were in categories detailing physical measures, blood assays and early life factors categories (Fig. 4c). Among all examined CNVs, the 22q11.2 deletion intermediate phenotype displayed the highest number of phenome-wide hits, with 90 robust associations after Bonferroni’s correction for multiple comparisons (Fig. 4c; for further details, see Supplementary Figs. 4–11). The collective results showed that CNVs are associated with numerous rich and diverse phenotypes across all 11 categories.

**Fig. 5:**
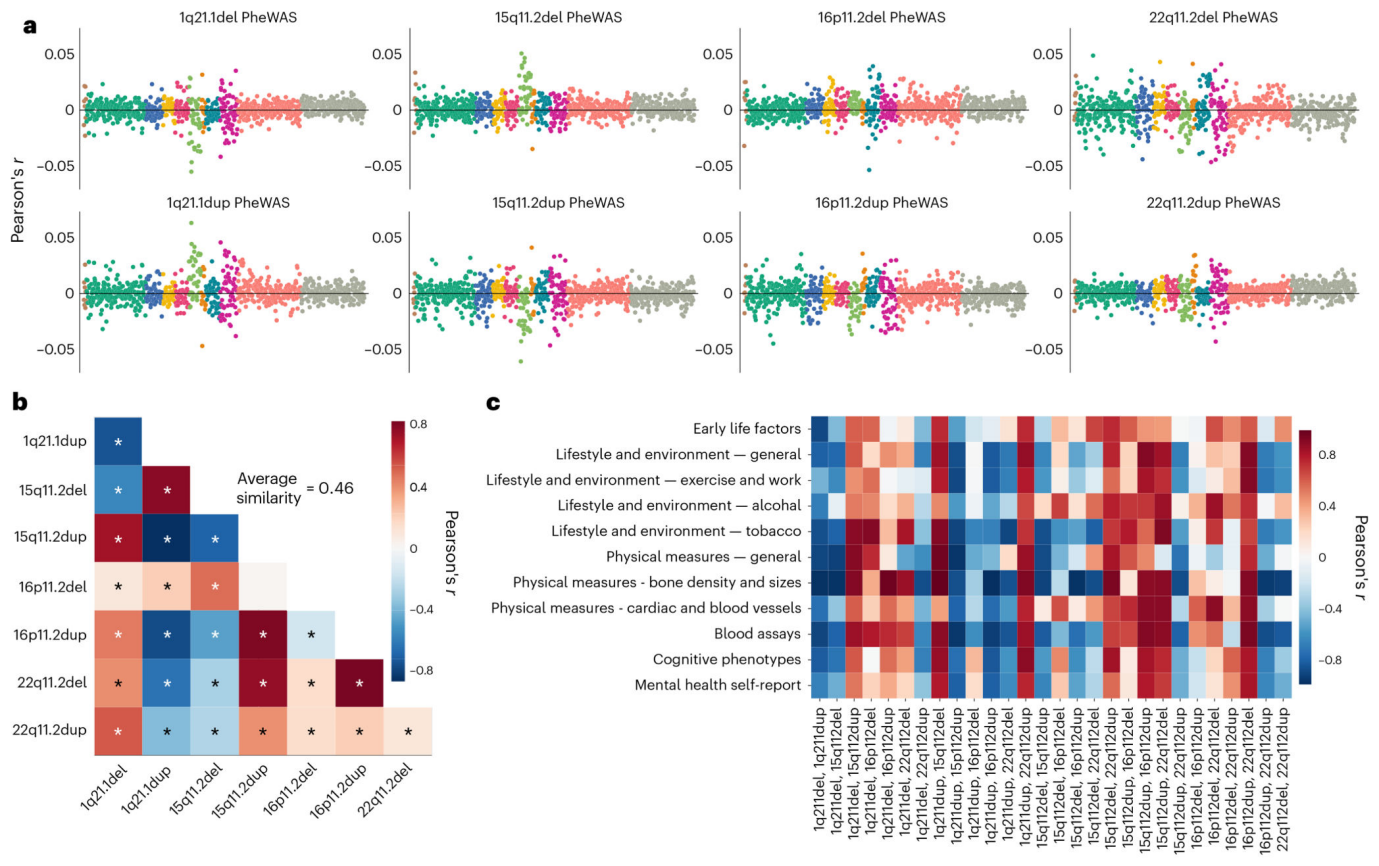
Eight different CNVs converge on similar phenome-wide association profiles. We carried out the PheWAS analysis for each intermediate phenotype to quantify the differences and commonalities in phenotypical consequences due to the 8 CNVs. **a**, Pearson’s correlations from PheWAS analysis for each CNV status. Among these, 22q11.2 deletion shows the strongest associations with numerous phenotypes across categories. Colours indicate the 11 categories. **b**, Linear association strength between PheWAS outcomes across all CNVs. Strong Pearson’s correlations suggest that CNVs are linked with similar phenotypes. Average similarity exceeds those of volumetric Cohen’s *d* maps and intermediate phenotypes. Asterisk denotes FDR-corrected significant correlations. **c**, Linear association strength between category-specific Pearson’s correlations from the PheWAS analysis across all CNVs. Detailed visualization depicts the similarity of the impact of CNVs on all phenotype categories. The direction of the linear relationship tends to be identical across categories for a given CNV pair (strong negative or strong positive), unlike across CNV pairs for a given category. The 8 CNVs exhibited similar PheWAS profiles, especially in bone density, blood assays and general physical measures categories.

Analogous to comparing volumetric signatures (cf. above), we examined the similarity of phenotypic profiles across CNVs. To this end, we calculated a correlation between the association profiles (Pearson’s correlations) from each PheWAS analysis (Fig. 5a). The definitive collection of brain signature-phenotype links reflected the linear association strength between CNV phenotypical profiles across 977 indicators (Fig. 5b). We found a strong resemblance (average similarity *r* = 0.62) among the 8 phenotypical profiles, with positive as well as negative correlations (Pearson’s correlations from *r* = −0.84 to 0.82). We subsequently zoomed in on the strong convergence across the phenotypic profiles characterizing each CNV by computing the correlation between CNV-phenotypic associations within each of the 11 considered categories (Fig. 5c). In particular, we found that bone density and sizes along with blood assay categories showed strong associations across CNV intermediate phenotypes, suggesting similar behaviour within these categories. Altogether, the strong correspondences among CNV pairs suggest that CNV brain profiles are linked to similar phenotypes across a rich portfolio of ~1,000 curated lifestyle indicators.

### Detailing shared and distinct phenotypic associations

**Fig. 6:**
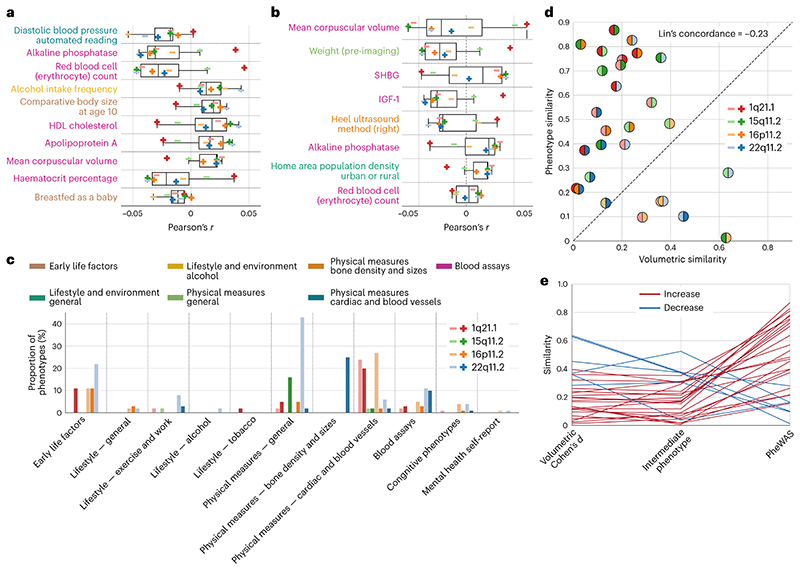
Detailing aspects convergence in phenome-wide portfolios across different CNVs. For all 8 CNVs, we delineate the most prominent as well as distinctive associations among their PheWAS profiles in 39,085 UK Biobank participants. We also compare CNVs on the basis of their brain and behaviour similarities. **a**, Phenotypes from the PheWAS analysis most strongly associated with the 8 CNVs. We show 10 phenotypes with the strongest average Pearson’s correlations across all CNVs. The most prominent association across CNVs is with diastolic blood pressure. The boxplot displays the first quartile, median, third quartile and whiskers corresponding to the appropriate quartile plus 1.5 times the interquartile range. **b**, Phenotypes most consistently associated with the 8 CNVs. We find 8 phenotypes associated with most (6) of the CNVs. Phenotypes are ordered according to the mean strength of the association. Most of the phenotypes are from the blood assays category. **c**, Number of significant hits per category for each intermediate phenotype conditioned on the shared phenotypical profile. For each of the 8 intermediate phenotype expressions, we regressed out the remaining 7. Even after conditioning on the shared phenotypical associations, each particular CNV still shows a specific set of distinct phenome-wide associations across various categories. For example, 22q11.2 deletion still displays a high number of associations in physical measures–general category. **d**, Concordance between brain volume effects and PheWAS effects. The absolute value of correlation between Cohen’s *d* brain maps (Fig. 1a) is plotted against the absolute value of correlation between PheWAS profiles. Negative Lin’s concordance correlation hints at the disparity between volumetric and phenotypical similarity. Moreover, the majority of points lie above the 45° line suggesting that PheWAS similarities are more substantial than volumetric similarities. **e**, From diverging brain patterns to converging portfolios. Each line represents a similarity of Cohen’s *d* map, intermediate phenotype and PheWAS profile for a given CNV pair. Convergence on PheWAS profiles is demonstrated by the increase in similarity in 22 of 28 CNV pairs. Hence, the similarity of CNV portfolios exceeded that of volumetric intermediate phenotypes.

To shed light on which particular phenotypes are most strongly associated with CNV-specific brain signatures, we calculated the mean absolute Pearson’s correlations across the 8 PheWAS analyses. Across all CNVs, diastolic blood pressure, alkaline phosphatase and red blood cell count showed the strongest associations (Fig. 6a). Moreover, we examined which phenotypes are most consistently associated with CNV brain profiles. We found 8 phenotypes associated with 6 CNV intermediate phenotypes and 11 phenotypes shared by 5 CNV intermediate phenotypes (Supplementary Fig. 3b,c). The most consistently overlapping phenotype hits were from the blood assays category (for example, sex hormone-binding globulin (SHBG), insulin-like growth factor 1 (IGF-1), mean corpuscular volume), along with weight or home population density. In total, these robust and shared phenotypic associations point to the fact that CNV brain profiles are associated with similar systemic phenotypes.

Comparisons of the phenotypical profiles associated with each CNV intermediate phenotype revealed that there remains unexplained residual variance, as suggested by a maximum absolute association strength of *r* = 0.81. To access this remaining part of the variance, we computed new brain profiles adjusting for the other CNVs. Specifically, for each CNV-specific intermediate phenotype, we singled out the variation explained by the remaining seven. Thus, we obtained a set of eight unique intermediate phenotypes, each with the variation shared with other intermediate phenotypes removed. Subsequently, we used this new set to perform the PheWAS analysis and counted the relative number of associations surpassing the Bonferroni correction in each category. We still observed significant associations across CNVs and categories even after conditioning out the shared associations. In particular, 22q11.2 deletion showed a high relative number of associations in the physical measures category (Fig. 6c). As such, next to the substantial phenotypic similarity, CNVs also displayed some unique characteristic phenotypic associations relative to other CNVs.

### Quantifying the path towards converging phenotypical profile

The observed magnitude of similarity between the phenotypic profiles of the CNV intermediate phenotypes, reaching Pearson’s *r* = 0.84, demonstrated a strong relationship between phenotypic profiles across the 977 indicators. In general, the phenotypic similarity (absolute Pearson’s correlation of PheWAS outcomes) between CNVs exceeded their morphological similarity (absolute Pearson’s correlation between Cohen’s *d* maps) (Fig. 6d). The dissonance between the two similarity measures was highlighted by Lin’s concordance correlation coefficient equal to -0.23, suggesting poor concordance. More specifically, 22 of 28 CNV pairs showed stronger phenotypical similarity compared with volumetric similarity. Thus, CNVs were characteristic of stronger phenotypic signature associations compared with associations among volumetric signatures or intermediate phenotypes (Fig. 6e).

Our collective analyses demonstrated that although each CNV displays largely distinct whole-brain morphometric signatures, they converged on similar phenotypic profiles. In proving this, we transferred the intermediate phenotypes derived in the clinical cohort to the UK Biobank population cohort with 39,085 participants. Using the participant-specific expression levels of 8 intermediate phenotypes from 8 rare CNVs allowed us to characterize complex phenotypical profiles of each CNV, providing a detailed portrait of their commonalities and idiosyncrasies.

## Discussion

CNVs offer a unique window of opportunity into the consequences of localized genetic variation on human traits. This is especially the case, given their known genetic architecture and typically high penetrance. In the present study, we built computational bridges between 8 key CNVs in a multisite clinical dataset on the one hand, and their deep phenotypic profiling in 39,085 participants from the wider population on the other hand. To this end, we designed an analytic framework that can quantitatively dissect the impact of distinct genetic mutations on brain organization and behavioural differentiation. Bringing over derived CNV-specific intermediate phenotypes to the population cohort revealed that the CNVs are tied to pleiotropic associations beyond physical and cognitive domains. This phenome-wide analysis across ~1,000 phenotypes revealed many ramifications for several body systems. Our collective analyses also reveal wide-ranging similarities between the PheWAS profiles of the 8 CNVs. Therefore, the phenotypic level appears to be the point of alignment for distinct long-segment genetic variants that we show to cause diverging morphological changes in brain morphology. Such late convergence in phenotypic consequences speaks to profound basic science questions regarding the organization of genetic influences on human brain and behaviour.

For a long time, inquiries targeting genetic influences have been limited by the lack of longitudinal and deep multimodal measures of brain and behaviour in large participant samples^24^. Studies aimed at elucidating genotype-phenotype links were challenged by several obstacles, including ascertainment bias, limited statistical power and patchy phenotypic coverage^22^. We are unlikely to have access to large enough clinical datasets soon–a condition sine qua non for definitive tests of phenotypic overlaps and differences between genetic variants. As a concrete example, ref. ^30^ highlighted the need for thousands of participants to obtain reproducible and reliable brain-wide associations. Therefore, to overcome several of these hurdles, we here put forward solutions that take advantage of intermediate CNV phenotypes, a term coined in research on psychiatric disorders^31^. These refer to biological traits that lie in between an individual’s external phenotype and innate genetic blueprint^32,33^. We captured CNV-specific intermediate phenotype representations as ‘genetics-first’ whole-brain signatures derived from our clinical boutique dataset. These signatures recapitulated previous findings on morphology alterations, such as the predominant decrease in regional volumes for deletions of 1q21.2 or 22q11.2, as well as the increase for 16p11.2 deletion^23,34,35^. We also observed reported mirror dose responses, especially strong in the 22q11.2 locus^22^. Therefore, the validity of LDA-derived intermediate phenotypes is corroborated by recapitulating key findings from clinical studies.

The 8 analysed CNVs are known to differ in the resulting effects on brain architecture^11,12^. The magnitude of their effects has previously been associated with the number of affected genes and clinical outcomes. In concordance, we found 16p11.2 deletion to affect the largest number of regions. This CNV contains 29 genes and is associated with an almost 40-fold increase in the odds of ASD^29^. Conversely, we found 15q11.2 duplication, which contains only 4 genes and is not formally associated with any disease, to affect the fewest number of regions. In addition, we provide a fresh look into the diverging CNV effects on brain morphology by summarizing the effects with respect to 7 large-scale Schaefer-Yeo networks. The network effects revealed a degree of similarity to functional connectivity alterations in CNV carriers^21^. We also observed effects in the default mode and limbic network for 22q11.2 deletion, as well as for ventral attention and motor network for 16p11.2 deletion. Together, the structural and functional alterations showed significant overlap with alterations in idiopathic ASD and SZ^36^. The resemblance suggests that the risks conferred by genetic variants, structural alterations and the associated functional connectivity patterns represent important dimensions that are coupled with diseases.

In the present work, we demonstrate the added value of how intermediate phenotypes can be transferred for direct usage in other cohorts, including large-scale populational datasets. By transferring these brain-wide representations over to the UK Biobank and carrying out PheWAS, we obtained systemic phenotypic associations across 11 rich phenotypic categories that go beyond mere cognitive domains. The reported PheWAS associations of the intermediate CNV phenotypes were concordant with previous studies investigating more circumscribed links between CNV status and indicators of cognitive performance, including fluid intelligence score^17^, physical measurements such as weight or height^3,15^, common medical conditions such as hypertension or obesity and blood biomarkers such as indicators of cholesterol fat metabolism pathways^37^. As one of many examples, we demonstrated how intermediate phenotypes tied to 22q11.2 deletion relate to an array of phenotypes in blood assays as well as cardiac and blood vessel categories. It is important to stress that PheWAS only charts associations between imaging and non-imaging measures to generate testable hypotheses without providing causal links^38^. Even though there may not be a direct causative link between a brain phenotype and cardiac biomarkers, the thus revealed association suggests a hidden causal effect of the CNV on both traits (for example, on brain morphology and artery wall thickening^39^).

Similar to ref. ^3^, 6 of our 8 examined CNVs were associated with body weight, IGF-1, alkaline phosphatase and mean red blood cell volume. Therefore, these bodily alterations may not be mere secondary effects^40^. Instead, systemic manifestations could be a fundamental aspect of the primary biology of CNVs and brain disorders in general. Critically, bodily manifestations might also lead to a reduced life span, as suggested by the 63% probability of survival to age 50 in adult carriers of 22q11.2 deletion^14,41^. Similar to 22q11.2 deletion, psychotic disorders have been linked with 15–20 yr shorter life expectancy^42^. Most of this premature mortality is predominantly due to elevated cardiovascular risk factors^43,44^–causes that belong to the phenotype category with the most consistent associations in our phenome-wide assays. Detected associations speak in favour of CNVs as a complex disorder with several manifestations outside the brain that have considerable deleterious impacts on various parts of everyday lives.

By combining hand-crafted analytic solutions with recently emerged data resources, our computational assays lay out pleiotropic associations in CNV carriers. These consequences include systemic associations outside the central nervous system. This underappreciated insight is reflected in our results, including strong brain-behaviour associations of the CNV profile in the UK Biobank population with blood pressure, cholesterol and weight. Since CNVs do not show complete penetrance in all cases^45^, such associations portray a necessary picture of a broad spectrum of outcomes later in life. Hence, the constellation of results advocates rebalancing the medical care of CNV carriers towards more comprehensive medical monitoring in a broader patient pool^46^.

In a similar way, previous clinical research has provided evidence that SZ and related psychotic disorders often affect multiple body systems (for example, nervous, immune or endocrine), even from illness onset^47,48^. Robust alterations in immune and cardiometabolic systems of a comparable magnitude to alterations in the central nervous system have been reported^40^. Further examples of major brain disorders accompanied by problems outside the brain include gastrointestinal disorders in autism^49^, loss of bone density in depression^50^ and cardiovascular symptoms in bipolar disorder^51^. Finally, a recent study showed that genetic liabilities for five major psychiatric disorders are associated with long-term outcomes in adult life, including sociodemographic factors and physical health^52^. Our findings thus add pieces of knowledge that illuminate how the nervous system is interlocked with the rest of the body in a way that affects general well-being.

More broadly, understanding pathophysiological disease mechanisms will be propelled by further disentangling the perplexing link between genes, brain and behaviour^53^. There is an active debate on the extent to which distinct gene dosage disorders can lead to different non-overlapping phenotypical profiles^24^. This discourse was sparked by the observations that many single nucleotide polymorphisms and CNVs increase the risk for SZ or autism^11,54^. Polygenicity and pleiotropy, key features of the genetics underpinning brain disorders^13,55^, imply that genetic mutations can converge on shared mechanisms at some level of pathway cascades, from genes to large-scale brain networks to the phenome. Here we report a low similarity of intermediate phenotypes representing morphological CNV-specific brain signatures, in line with a documented broad diversity of regional morphometry patterns across genomic loci^22,56,57^. Conversely, the ramifications of carrying distinct CNV variants for cognition and behaviour have previously been hypothesized to be more similar than those of brain anatomy ^9,24^. We here find evidence for substantial convergence of phenotypic measures across CNVs quantified by increased phenotypical similarity. Specifically, we observed a high degree of similarity between the phenotypical profiles (mean similarity *r* = 0.46 as measured by Pearson’s correlation across the CNV’s corresponding PheWAS profiles), which largely exceeded the similarity of brain morphometry profiles (mean similarity *r* = 0.2 as measured by the correlation of volumetric Cohen’s *d* maps). Based on the presented strong resemblance of phenotypic profiles of the examined 8 CNVs, we speculate that the polygenic architecture of human phenotypic traits may be more related to genotype-phenotype convergence that occurs later than on molecular pathways or macroscopic brain networks.

This study has several limitations. One is that we did not investigate the effects of medication on derived CNV-specific brain signatures since medication information was not available for the whole clinical dataset. Nevertheless, previous studies have reported no significant effects of psychiatric comorbidities (for example, psychosis, ASD, attention-deficit/hyperactivity disorder (ADHD), anxiety and mood disorder) and psychotropic medication on neuroimaging patterns^36,58^. We also did not study causal relationships between brain patterns and non-imaging indicators. Making causal inference requires proposing and defending a plausible causal structure by spelling out the assumed (directional) dependencies among the outcome, input variables and relevant confounding variables^59^. Future studies can start off from hypotheses generated by our catalogue of PheWAS links to find causal links between variables, for example, using structural equation modelling. Finally, given our data scenario (cf. ^26,27^, we resorted to linear models in combination with bagging and shrinkage to safeguard from overfitting.

In conclusion, we have triangulated (1) a purpose-designed analytical strategy, (2) a roadmap for investigating rare brain pathologies employing intermediate phenotypes derived from smaller clinical datasets and (3) a framework for application in population-scale cohorts. Our results highlight the potential of using intermediate phenotypes as a device to study a wide variety of rare conditions and thus accelerate the pace of neurogenetic innovation. By building bridges between the broad population of the UK Biobank and carefully collected clinical datasets, we derived prediction models for CNV-specific brain phenotype expressions that can be used in other hospitals and healthcare institutions. Deep phenotypic profiling of these models clearly demonstrates that CNVs may have whole-body manifestations. Therefore, our study shows that CNV effects go beyond relevance for childcare and psychiatry by potentially extending to other areas of medical care and treatment, which are blind spotted today. In addition, detected overlapping system-wide phenotype associations across multiple CNVs advance our understanding of genotype-phenotype correspondences.

## Methods

### Multisite clinical cohort

Signed consents were obtained from all clinical participants or legal representatives before the investigation. The current study, which is purely analytical, was approved by the institutional review boards (Project 4165) of the Sainte Justine Hospital. UK Biobank participants gave written, informed consent for the study, which was approved by the Research Ethics Committee. The current analyses were conducted under UK Biobank application number 25163. Further information on the consent procedure can be found online (biobank.ctsu.ox.ac.uk/ crystal/field.cgi?id=200).

Our clinical dataset consisted of volumetric measurements derived from MRI brain scans of 860 participants: 548 CNV carriers and 312 controls not carrying any CNV (Table 1). The here examined CNVs are among the most commonly studied CNVs^60^. Deletions and duplications of 1q21.1, 15q11.2, 16p11.2 and 22q11.2 represent some of the most frequent risk factors for neuropsychiatric disorders identified in paediatric clinics^19,20^. That is why the target CNV loci were also selected by the Enhancing NeuroImaging Genetics through Meta-Analysis copy number variant (ENIGMA-CNV) in a study on their cognitive, psychiatric and behavioural manifestations^23^. These deletions and duplications strike a balance between occurrence in the population and their effect size. In other words, the selected CNVs are frequent enough so that we can start studying large enough sample sizes that allow for across-CNV comparison in the first place. At the same time, this class of CNVs has been shown to detrimentally affect cognition and raise the risk for psychiatric conditions^23,25,35^. Our CNV carriers did not carry any other large CNV.

An extensive description of methods and analyses is available in an already published study with an identical dataset^60^. In short, PennCNV and QuantiSNP were used, with standard quality control metrics, to identify CNVs. CNV carriers were selected on the basis of the following breakpoints according to the reference genome GRCh37/hg19: 16p11.2 proximal (BP4-5, 29.6-30.2MB), 1q21.1 distal (Class I, 146.4-147.5MB & II, 145.3-147.5MB), 22q11.2 proximal (BPA-D, 18.8-21.7MB) and 15q11.2 (BP1-2, 22.8–23.0MB). Control individuals did not carry any CNV at these loci. The CNV carriers were either probands referred to the genetic clinic for the investigation of neurodevelopmental and psychiatric disorders or their relatives (parents, siblings and other relatives).

UK Biobank might represent the largest dataset of carriers affected by 15q11.2 deletions and duplications. Therefore, after identifying 15q11.2 deletions and duplications in the UK Biobank, we added the respective carriers to our clinical cohort. In other words, we excluded these participants from the UK Biobank and treated them as part of our clinical dataset. Sensitivity analysis concluded that including this CNV locus did not change our main findings (Supplementary Fig. 12). Controls were either non-carriers within the same families or individuals from the general population. Furthermore, controls were carefully matched for sex and age to CNV carriers.

### Clinical MRI data recording and processing

We analysed a data sample of T1-weighted (T1w) images at 0.8–1 mm isotropic resolution. All T1w images included in the analysis were quality checked by a domain expert^60^. Data for voxel-based morphometry were preprocessed and analysed with SPM12 (http://www.fil.ion.ucl.ac.uk/spm/software/spm12/)^61–63^ running under MATLAB R2018b (https://www.mathworks.com/products/new_products/release2018b.html). Further quality control was performed using standardized ENIGMA quality control procedures (http://enigma.ini.usc.edu/protocols/imaging-protocols/). Finally, neurobiologically interpretable measures of grey matter volume were extracted for all participants by summarizing whole-brain MRI maps in the MNI reference space. This feature-generation step was guided by the topographical brain region definitions of the commonly used Schaefer-Yeo atlas with 400 parcels^64^. The derived quantities of local grey matter volumetry resulted in 400 volume measures for each participant. As a data-cleaning step, derived regional brain volumes were adjusted for intracranial volume, age, age^2^ and sex as fixed effects and scanning site as a random factor, following previous research on this dataset^60^. In particular, we have previously demonstrated that CNVs show independent effects on regional and total brain volumes^35^. Our current investigation is focused on how CNVs induce regional brain effects. Note that ancillary analyses revealed additional adjustments for total grey matter volume not to have any appreciable effect on subsequent analyses.

### Population data source

The UK Biobank is the largest biomedical resource that offers extensive behavioural and demographic assessments, medical and cognitive measures, as well as biological samples in a cohort of ~500,000 participants recruited from across Great Britain (https://www.ukbiobank.ac.uk/). The present study was based on the recent brain-imaging data release from February/March 2020. Our data sample included measurements from 39,085 participants with brain-imaging measures and expert-curated image-derived phenotypes of grey matter morphology (T1w MRI) (Table 2). Among the participants, 48% were men and 52% were women, with age between 40 and 69 yr old when recruited (mean age ±s.d.: 55 ± 7.5yr). We benefited from the uniform data preprocessing pipelines designed and implemented by the FMRIB, Oxford University, UK^65^, to improve comparability and reproducibility.

MRI scanners (3T Siemens Skyra) at several dedicated data collection sites used matching acquisition protocols and standard Siemens 32-channel radiofrequency receiver head coils. Brain-imaging measures were defaced to protect the study participants’ anonymity and any sensitive meta-information was removed. Automated processing and quality control pipelines were deployed^38,65^. To improve the homogeneity of the brain-imaging scans, the noise was removed using 190 sensitivity features. This approach allowed for the reliable identification and exclusion of problematic brain scans, such as those due to excessive head motion.

The structural MRI data were acquired as high-resolution T1w images of brain anatomy using a 3D MPRAGE sequence at 1 mm isotropic resolution. Preprocessing included gradient distortion correction, field of view reduction using the Brain Extraction Tool^66^ and FLIRT^67^, as well as nonlinear registration to MNI152 standard space at 1 mm resolution using FNIRT^68^. To avoid unnecessary interpolation, all image transformations were estimated, combined and applied by a single interpolation step. Tissue-type segmentation into the cerebrospinal fluid, grey matter and white matter to generate full bias-field-corrected images was achieved using FAST (FMRIB’s Automated Segmentation Tool^69^). Finally, grey matter images were used to extract grey matter volumes in parcels according to the Schaefer-Yeo atlas with 400 regions^64^. Following previous work on the UK Biobank^70,71^, interindividual variations in brain region volumes that could be explained by nuisance variables of no interest were adjusted for by regressing out: body mass index, head size, head motion during task-related brain scans, head motion during resting-state fMRI scanning, head position and receiver coil in the scanner (*x*,*y* and *z*), position of the scanner table, as well as the data acquisition site.

### Statistical analysis for volumetric brain measures

All subsequent analyses were performed in Python v3.8 as a scientific computing engine (https://www.python.org/downloads/release/python-380/). We used Cohen’s *d* to quantify the effect size of the CNVs on individual regional volumes. For a given region, Cohen’s *d* is defined as:

$$d=\frac{\bar{x_{1}}-\bar{x_{2}}}{\sqrt{\frac{s_{1}^{2}+s_{2}^{2}}{2}}},$$

where $\bar{x_{1}}$ corresponds to the mean region volume across CNV carriers, $\bar{x_{2}}$ corresponds to the mean region volume across controls. Similarly, *s*_1_ and *s_2_* correspond to standard deviations of CNV carriers and controls.

Results from Cohen’s *d* analyses were confirmed by a nonparametric effect size measure (Supplementary Fig. 13).

We compared Cohen’s *d* volumetric brain maps (and intermediate phenotype brain maps) between different CNVs using Pearson’s correlation. Furthermore, we used spin permutation testing with 1,000 iterations to calculate empirical *P* values for the resulting correlation coefficient^72^.

Finally, we calculated Lin’s concordance correlation coefficient to quantify the agreement of similarities between volumetric Cohen’s *d* maps, intermediate phenotypes and PheWAS profiles. The degree of concordance between the two measures is thus calculated as:

$$\rho_{\text{C}}=\frac{2s_{1,2}}{s_{1}^{2}+s_{2}^{2}+{\left(\bar{x_{1}}-\bar{x_{2}}\right)}^{2}},$$

where s_1,2_ corresponds to the covariance between *x*_1_ and *x*_2_.

### Charting complex association using PheWAS

We performed a rich annotation of the derived intermediate phenotypes by means of a phenome-wide association analysis benefitting from a wide variety of almost 1,000 lifestyle factors. For a detailed description of phenotype extraction and analysis, refer to our previously published studies^73^. Feature extraction was carried out using two utilities designed to obtain, clean and normalize UK Biobank phenotype data according to predefined rules. Briefly, we collected a raw set of -15,000 phenotypes that we further processed by the FMRIB UKB normalization, parsing and cleaning kit (FUNPACK v2.5.0; https://zenodo.org/record/4762700#.YQrpui2caJ8). FUNPACK is designed to perform automatic refinement on the UKB data, which includes removing ‘do not know’ responses and filling the blank left by unanswered sub-questions. The FUNPACK-derived phenotype information covered 11 major categories, including cognitive and physiological assessments, physical and mental health records, blood assays, as well as sociodemographic and lifestyle factors. The output consisted of a collection of 3,330 curated phenotypes which were then fed into the PHEnome Scan ANalysis Tool (PHESANT^74^, https://github.com/MRCIEU/PHESANT) for further refinement in an automated fashion. PHESANT performs further data cleaning and normalization along with labelling data as one of four data types: categorical ordered, categorical unordered, binary and numerical. Categorical unordered variables were one-hot encoded, such that each possible response was represented by a binary column (true or false). The final curated inventory comprised 977 phenotypes spanning 11 FUNPACK-defined categories. Furthermore, we used Pearson’s correlation to quantify the association strength between these 977 phenotypes and participant-specific expressions of our 8 intermediate phenotypes (cf. below). To ensure that the correlations are not driven by a few outlying intermediate phenotype expressions, we first discarded 551 participants on the basis of Tukey’s interquartile range rule for outlier detection.

### Multiclass prediction model and intermediate phenotype extraction

Technically, our core aim was to derive robust CNV-specific representations of intermediate phenotypes from a clinical sample that could be transferred to a large population resource for deep profiling. We derived the intermediate phenotypes as systematic brain morphometric co-deviations attributable to each of our 8 target CNVs. To this end, we capitalized on LDA to extract separating rules between CNV carriers and controls on the basis of whole-brain volume measurements. LDA can be viewed as a generative approach to classifying CNV carriers, which requires fitting a multivariate Gaussian distribution to regional brain volumes and producing a linear decision boundary^75^. In particular, LDA-derived discriminant vectors/functions represented CNV-specific intermediate phenotypes. Using a linear model represents a data-efficient and directly biologically interpretable approach to our analysis, especially in our boutique datasets with limited participant samples^76^. These datasets are characteristic of the low sample size regularly encountered in biology and medicine, which typically impedes the application of more complex nonlinear models that require high numbers of parameters to be estimated.

As another key model property of direct relevance to our present analysis goals, LDA can also be viewed as a dimensionality technique because this modelling framework enables the extraction of underlying coherent principles among our anatomical target regions that are most informative in telling apart CNV carriers from controls. To do so, LDA has access to class labels (CNV status in our case) and thus belongs to supervised techniques^75^. Specifically, LDA projects the input participants’ set of brain morphology measurements into a linear subspace, consisting of the directions which maximally separate our classes^77^. This dimensionality reduction quality of LDA was a prerequisite for extracting intermediate phenotypes from one dataset and transferring them to other datasets.

In our study, we used LDA models to classify between CNV carriers and controls. Specifically, we derived a single LDA prototype for each CNV status, which yielded 8 CNV-specific models. The dimensionality reduction capability of the LDA framework provides biologically interpretable compact views on distinguishing the CNV carriers from controls on the basis of a linear combination of brain region volumes. As a general rule, the maximum number of dimensions equals the number of classes minus 1. Since each LDA model instance discriminated between two classes at hand (for example, controls and 22q11.2 deletion), we obtained a one-dimensional vector encapsulating the 22q11.2 deletion intermediate phenotype. This vector of coefficients revealed the concomitant contribution of each brain region volume towards the separability of the CNV carriers on the basis of whole-brain morphology measurements. Therefore, the coefficients provided quantitative information on the relative importance of the collective brain regions for CNV-health separation. Moreover, the LDA coefficients were estimated together with the other brain region volume effects, in contrast to the estimation of marginal or partial variable effects as in linear regression. Furthermore, to embed each participant’s brain morphology in a low-rank subspace that maximally separates 22q11.2 deletion carriers and controls, we used the LDA coefficient vector to re-express (that is, project) the set of 400 regional volumes of a given participant onto a single dimension representing 22q11.2 deletion intermediate expression level signature. Finally, as a step from dimensionality reduction to classification, these expressions of predictive participant brain morphology indicators were then used to construct a discriminant function.

### Building and validating robust prediction models

To recapitulate, our goal was to derive 8 CNV-specific intermediate phenotypes using LDA. Therefore, we built separate CNV-specific LDA models designated to learn predictive principles to distinguish CNV carriers from controls. However, we faced the challenge of the low number of CNV carriers. This challenge is inherent to various boutique datasets of rare medical conditions. Consequently, our number of measured features (regional brain volumes) was higher than the number of observation samples (participants). Concretely, we disposed on average 67 participants per CNV class (cf. Table 1), while each participant was described by 400 regional volumes. Such a high-dimensional data scenario can lead to overfitting^26^, where the model learns the detail and noise in the training samples and performs poorly in group classification on unseen test samples^75^. Hence, we used bootstrap aggregation (bagging), an ensemble learning method that can be used to reduce overfitting^78^. Bagging gains its value by profiting from a wisdom-of-crowds strategy. Concretely, we used a set of trained LDA models to obtain a more robust and better predictive performance than could be obtained from a single trained LDA model in isolation^78^. Such a model-averaging design improves classification performance by reducing variance^75^.

We performed bagging during the derivation of LDA models separately for all 8 CNV classes. Specifically, we used the following analytical strategy for a set of participants consisting of a single CNV type and controls. In the first phase, a randomly perturbed version of the dataset was created by sampling the participant cohort with replacement. This bootstrap resampling served as the ‘in-the-bag’ set of samples (that is, participants). The number of ‘in-the-bag’ CNV carriers and controls equalled their number in the dataset. Furthermore, the LDA model was trained on this training ‘in-the-bag’ dataset. Model performance was then evaluated on all participants from the dataset that were not selected for the ‘in-the-bag’ dataset. These participant samples formed a testing ‘out-of-bag’ dataset. The performance (that is, classification accuracy) was based on the Matthews correlation coefficient, which has been reported to produce a more informative and truthful score than accuracy and the F1 score^79^. The coefficient ranges between −1 and +1, where a coefficient of +1 represents a perfect prediction, 0 a random prediction and -1 indicates total disagreement between prediction and observation.

We repeated the bootstrap resampling procedure with 100 iterations. In so doing, we obtained different realizations of the entire analysis process and the resulting LDA model estimate. Concretely, the bagging algorithm resulted in 100 trained LDA models used to obtain 100 out-of-bag predictions in unseen participants. We calculated the final prediction accuracy as a mean across the 100 performance estimates. Critically, the average over the collection of separately estimated LDA discriminant functions served as our CNV-specific intermediate phenotype that provided the basis for downstream analysis steps. Finally, we characterized each participant by the intermediate phenotype expression level, which we calculated as the average one-dimensional LDA projection of regional volume sets across the 100 replications. In summary, the variance of local information in the 100 redraws of our original clinical participant cohort promoted diversity among the obtained candidate predictive rules, thus strengthening the fidelity of our ultimate predictions.

To further safeguard against the risk of overfitting, we optimized the shrinkage parameter of each LDA model. Shrinkage corresponds to regularization used to stabilize the estimation of model parameters, such as in covariance matrices during model training. The empirical sample covariance is a poor estimator when the number of samples is small compared with the number of features. The covariance matrix estimation involved an interpolation between the sample covariance matrix based on the maximum likelihood estimator and a weighted identity matrix, which amounted to the l2-penalization of the covariance matrix that then provided the basis for deriving a robust LDA solution.

Indeed, our sample covariance matrix held 80,200 unique entries, almost 1,200 times more than the average number of CNV carriers available. Therefore, the vanilla estimation of the covariance matrix is singular and thus degenerates for downstream analysis steps, such as matrix inversion. To avoid such an inversion problem, we applied a dedicated shrinkage approach for the covariance matrix estimation step within LDA (ShrunkCovariance function from ‘sklearn’). Using a nested cross-validation architecture, we performed a rigorous search over 11 shrinkage hyperparameter choices between 0 and 1, in steps of 0.1, in each ‘in-the-bag’ bootstrap iteration (GridSearchCV function from ‘sklearn’). The optimal hyperparameter choice was based on a leave-one-out strategy. In this cross-validation technique, each sample of the ‘in-the-bag’ dataset was used once as a test set of unseen participants, while the remaining participant samples formed the training set.

Finally, we evaluated the significance of a cross-validated score and thus assessed whether our ensemble LDA model displayed above-chance classification performance. Specifically, we carried out a label permutation test to quantify whether our LDA model outperforms the empirical null model. The null distribution was generated by calculating the prediction accuracy of our LDA classifier on 100 different permutations of the dataset. In these, features remained unchanged, but class labels (that is, CNV carriers or controls) were randomly shuffled. Such a shuffling corresponded to the null hypothesis, which states no dependency between the features and labels. The LDA model displayed above-chance classification performance if its prediction accuracy was higher than the 97.5th percentile of the prediction accuracy coefficient distribution derived from 100 permuted models.

### Performing model inspection using feature importance

After deriving robust LDA classifiers, we inspected which brain regions were the most informative in distinguishing CNV carriers from controls. In other words, we aimed to contextualize and unpack the prediction rules of our ensemble LDA model. The bagging algorithm led to obtaining a collection of LDA models, resulting in a collection of estimates for each LDA coefficient and participant-specific intermediate phenotype expressions. Since each LDA model is trained on a different bootstrap population, it might happen that two distinct LDA models’ coefficients would carry opposite signs due to the sign invariance of LDA dimensionality reduction. Therefore, we aligned all LDA models by multiplying them with −1 or 1 to produce a positive correlation between LDA coefficients and a corresponding Cohen’s *d* map.

Furthermore, we designed a criterion to test which LDA coefficients are significant, meaning which features significantly contribute to the classification. Significant coefficients had the distribution of 100 LDA coefficients significantly different from 0. Specifically, they were robustly different from zero if their two-sided confidence interval according to the 2.5/97.5% bootstrap-derived distribution did not include zero.

### Carrying intermediate phenotype expressions over for deep characterization in other data resources

One of the aims of this study is to use a population dataset to investigate derived intermediate phenotypes. To do so, we transferred the CNV-specific intermediate phenotypes carefully derived in our boutique dataset and quantified their expression in the general population (that is, UK Biobank). It is important to note that the derived intermediate phenotypes were not influenced by ASD or SZ diagnosis (Supplementary Fig. 14).

UK Biobank itself contains CNV carriers. Therefore, we aimed to validate the transferability of intermediate phenotypes by testing the difference in intermediate phenotype expression between CNV carriers and controls in both the clinical dataset and UK Biobank. Specifically, we tested the null hypothesis of no difference in the mean expression of intermediate phenotype in CNV carriers and controls. We adopted a two-sample bootstrap hypothesis test for means difference with 1,000 bootstrap replicates^80^.

### Reporting summary

Further information on research design is available in the Nature Portfolio Reporting Summary linked to this article.

## Supplementary Material

Supplementary Material

## Figures and Tables

**Table 1:** Clinical dataset demographics

Loci	Chr (hg19) start-stop	nGenes (Gene)	Type	Participants	Age (s.d.)	Sex (M/F)	ASD|SZ diagnosis	Other diagnoses
1q21.1	chr1	7	Del	24	31 (18)	9 / 15	0|0	4
	146.53-147.39	CHDIL	Dup	15	33 (17)	7 / 8	3|0	2
15q11.2	chr15	4	Del	112	55 (7)	51 / 61	0|0	2
	22.81-23.09	CYFIP1	Dup	146	54 (7)	69 / 77	0|0	6
16p11.2	chr16	27	Del	80	17 (12)	46 / 34	10|0	10
	29.65-30.20	KCTD13	Dup	69	31 (14)	37 / 32	7|1	10
22q11.2	chr22	49	Del	69	17 (9)	33 / 36	8|2	29
	19.04-21.47	AIFM3	Dup	19	19 (14)	12 / 7	2|0	5
Controls				312	26 (14)	179/133	1|0	12

CNV loci chromosome coordinates are provided with the number of genes encompassed in each CNV and with a well-known gene for each locus to help recognize the CNV. Other diagnoses included: language disorder, major depressive disorder, posttraumatic stress disorder, unspecified disruptive and impulse-control and conduct disorder, social anxiety disorder, social phobia disorder, speech sound disorder, moderate intellectual disability, specific learning disorder, gambling disorder, bipolar disorder, conduct disorder, ADHD, substance abuse disorder, global developmental delay, motor disorder, obsessive-compulsive disorder, sleep disorder, Tourette’s disorder, mood disorder, eating disorders, transient tic disorder, trichotillomania, pervasive developmental disorder, specific phobia, body dysmorphic disorder, mathematics disorder and dysthymic disorder. Del: deletion; Dup: duplication; Chr: chromosome; Age: mean age; nGenes: number of genes.

**Table 2:** UK Biobank imaging demographics

	Non-carriers	1q21.1 del		15p11.2 del		16p11.2 del		22q11.2 del	
		del	dup	del	dup	del	dup	del	dup
Participants	38,731	12	14	117	155	4	7	5	47
Percent female	52	42	64	54	53	25	43	60	43
Age (s.d.)	55 (8)	51 (6)	54 (7)	55 (7)	54 (7)	58 (3)	55 (6)	53 (8)	54(8)
ASD^a^|SZ^b^ diagnosis	68|18	0|0	0|0	0|0	0|0	0|0	0|0	0|0	0|0

Our data sample included measurements from 39,085 participants with brain-imaging measures and expert-curated image-derived phenotype. Based on the cohort’s sociodemographic, physical, lifestyle and health-related characteristics, UK Biobank participants are known to be close to the general population^81^. CNVs were identified using PennCNV and QuantiSNP. UK Biobank might represent the largest dataset of carriers affected by 15q11.2 deletions and duplications. Therefore, we excluded these participants from the UK Biobank and treated them as part of our clinical dataset. The remaining CNV carriers served for validation of derived LDA prediction patterns. ^a^ICD10 code, including diagnoses of SZ, schizotypal and delusional disorders (F20-F29). ^b^ICD10 code, including diagnoses of childhood autism (F84.0), atypical autism (F84.1), Asperger’s syndrome (F84.5), other pervasive developmental disorders (F84.8) and pervasive developmental disorder, unspecified (F84.9). Mean age is depicted along with the standard deviation.

## Data Availability

The majority of 16p11.2 data are publicly available (https://www.sfan.org/). For the 22q11.2 sample, raw data are available upon request from the PI (C.E.B., cbearden@mednet.ucla.edu). All derived measures used in this study are available upon request (S.J., sebastien.jacquemont@ umontreal.ca). The rest of the CNV carriers’ data cannot be shared as participants did not provide consent. All data from UK Biobank are available to other investigators online (ukbiobank.ac.uk). The Schaefer-Yeo atlas is accessible online (https://github.com/Thom-asYeoLab/CBIG/tree/master/stable_projects/brain_parcellation/Schaefer2018_LocalGlobal). Source data are provided with this paper.

## Code availability

The processing scripts and custom analysis software used in this work are available in a publicly accessible GitHub repository along with examples of key visualizations in the paper: https://github.com/dblabs-mcgill-mila/CNV-convergence.
