# Supplementary material for: Rare CNVs and phenome-wide profiling highlight brain structural divergence and phenotypical convergence
Source: Nat Hum Behav. Author manuscript; Available in PMC 2023 Nov 9. (PMC7615290; doi:10.1038/s41562-023-01541-9)
Supplement: Supplementary Material [file EMS190340-supplement-Supplementary_Material.pdf]

nature human behaviour

Article

https://doi.org/10.1038/s41562-023-01541-9

Rare CNVs and phenome-wide profiling highlight brain structural divergence and phenotypical convergence

In the format provided by the authors and unedited

Supplementary Figures

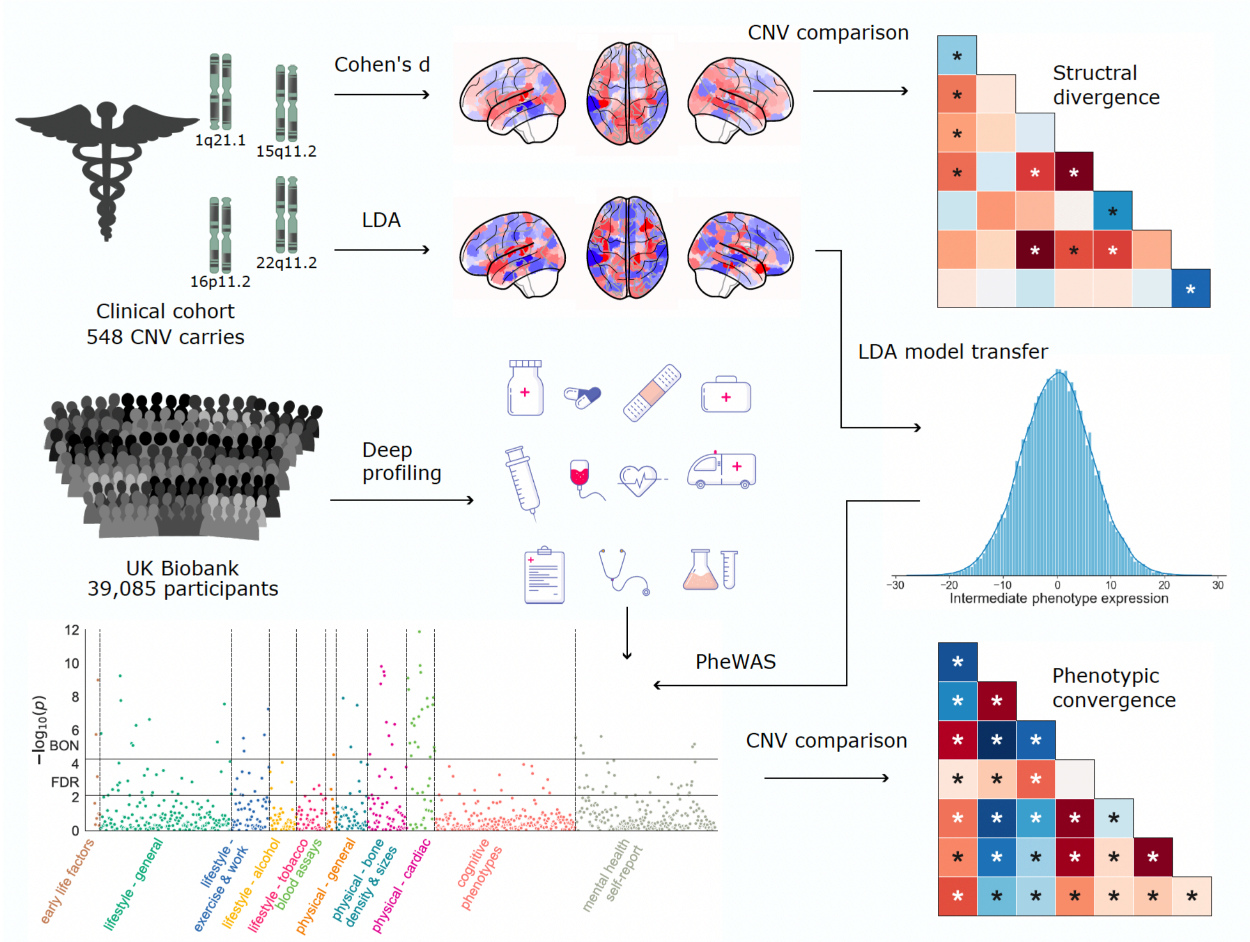

<u>Supplementary Figure 1</u>

Schematic for analysis workflow.

In 534 subjects carrying one of eight CNVs, we have computed Cohen's d between the carriers and 312 controls separately for each of the 400 brain regions (Schaefer-Yeo reference atlas). Cohen's d brain map quantifies the magnitude of structural change for each CNV. The low magnitude of Pearson's correlations between Cohen's d brain maps of different CNVs shows that CNVs have distinct effects on brain volumes. In the next step, we estimated eight LDA models to classify between controls and each of the eight different CNVs. We then performed a phenome-wide association study (PheWAS) by computing Pearson's correlation between the expression of each of the eight intermediate CNV phenotypes and 977 phenotypes spanning 11 categories in 39,085 UK Biobank subjects. When we compared PheWAS profiles across CNVs, we found strong correlations suggesting that CNVs are linked with similar phenotypes. In conclusion, the similarity of CNV phenotypic portfolios exceeded those of volumetric intermediate phenotypes.

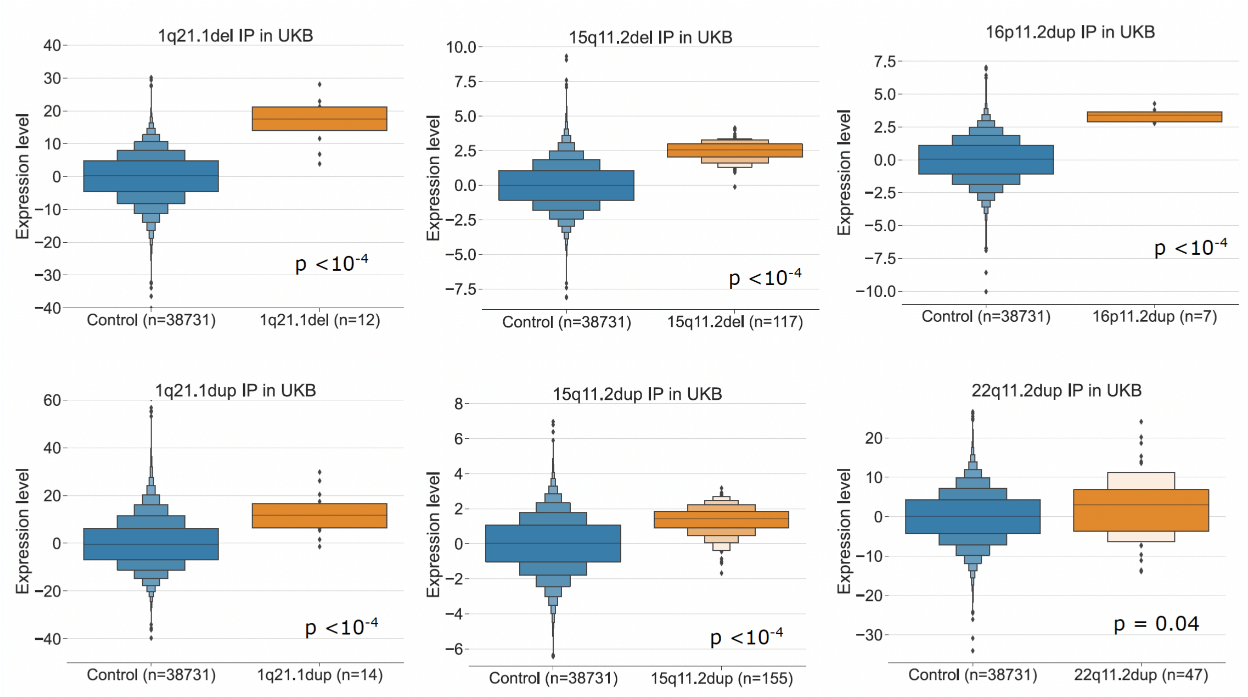

<u>Supplementary Figure 2</u>

Intermediate phenotype expressions in the UK Biobank

We quantified the presence of each CNV-specific intermediate phenotype derived from the clinical dataset. Here, we plot letter-value (boxen) plots for CNVs with at least five carriers in the UK Biobank. In all of these CNVs, the expression of respective intermediate phenotype was higher in actual CNV carries than in non-CNV carries based on two-sample bootstrap hypothesis test for difference of means with 10,000 bootstrap replicates. The boxen plot depicts a large number of quantiles for the expression scores.

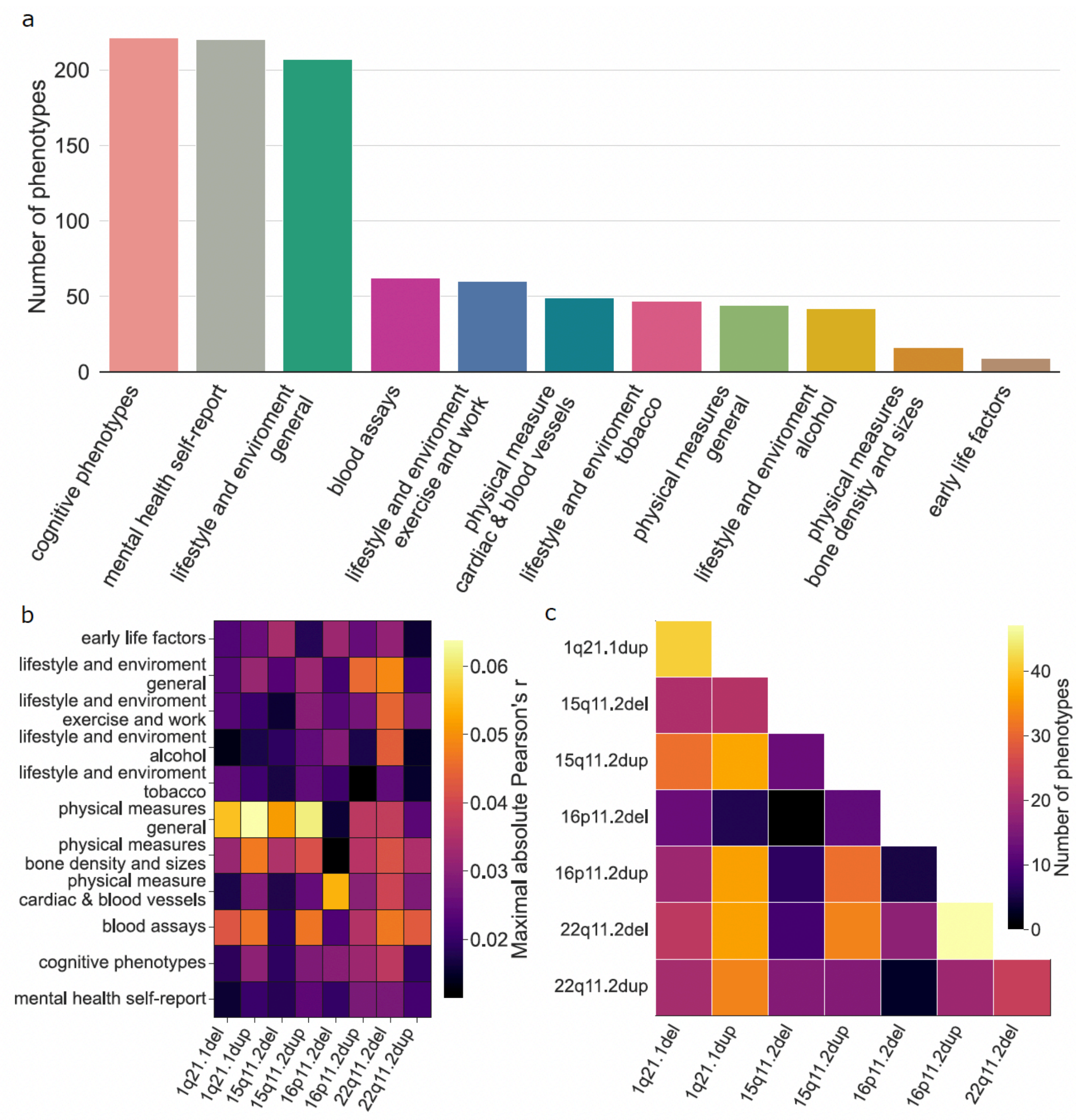

Supplementary Figure 3

Dissecting PheWAS analyses across 11 categories

We inspected which of our eleven categories are most strongly and most consistently associated with CNV status. a) The 977 available phenotypes span eleven categories. Three categories dominated our PheWAS, with more than 200 phenotypes per category. b) Strongest associations across categories and CNVs. We plot the maximal absolute Pearson's correlation for each category and CNV. The strongest associations are in the physical measures – general and blood assays category. c) Shared associations across CNVs. The number of shared phenotypical associations varied between 0 and 43 (16p11.2 proximal duplication and 22q11.2 deletion). Moreover, the number of shared associations is significantly correlated with the phenotypical similarity between CNVs ($r = 0.80$, $p < 10^{-4}$). These results support the high phenotypical similarities among different CNV loci.

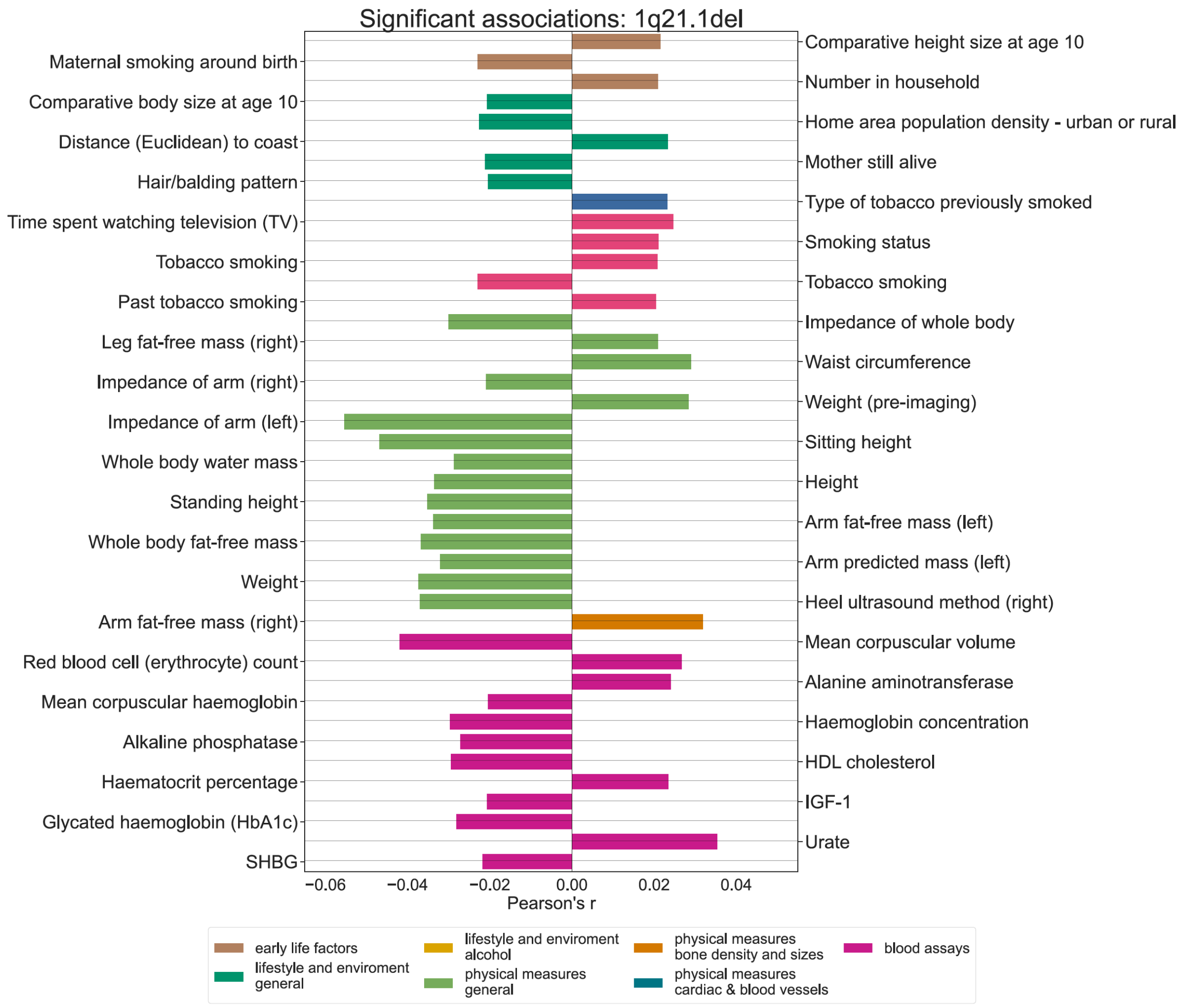

Supplementary Figure 4

Significant PheWAS associations for 1q21.1 distal deletion intermediate phenotype expression.

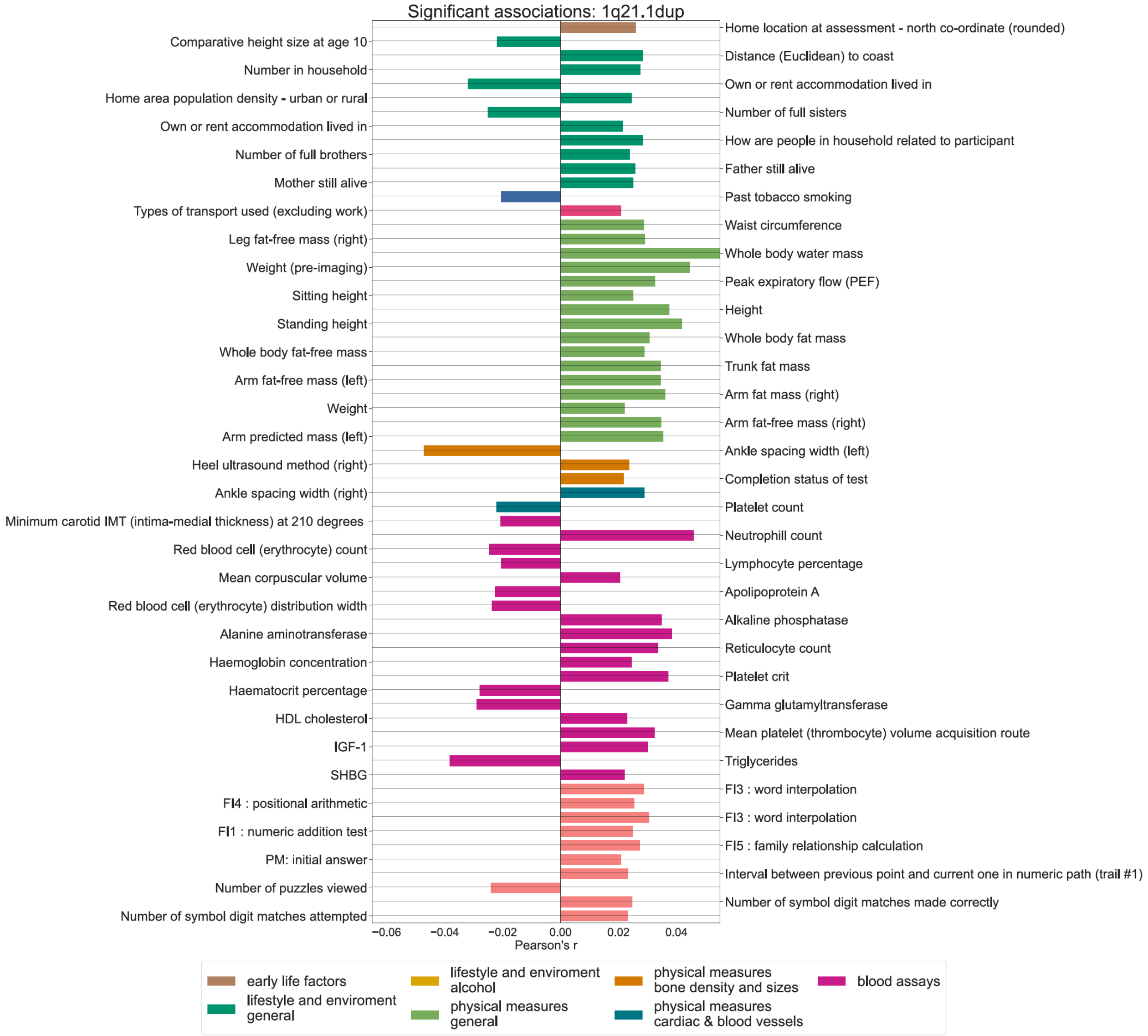

Supplementary Figure 5

Significant PheWAS associations for 1q21.1 distal duplication intermediate phenotype expression.

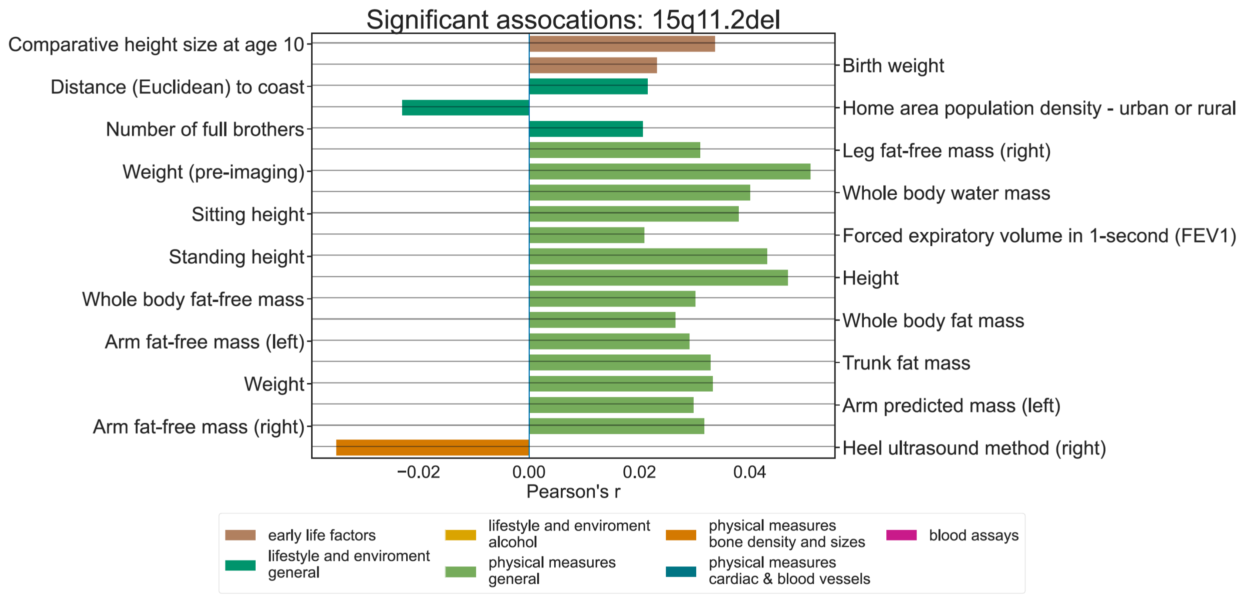

Supplementary Figure 6

Significant PheWAS associations for 15q11.2 deletion intermediate phenotype expression.

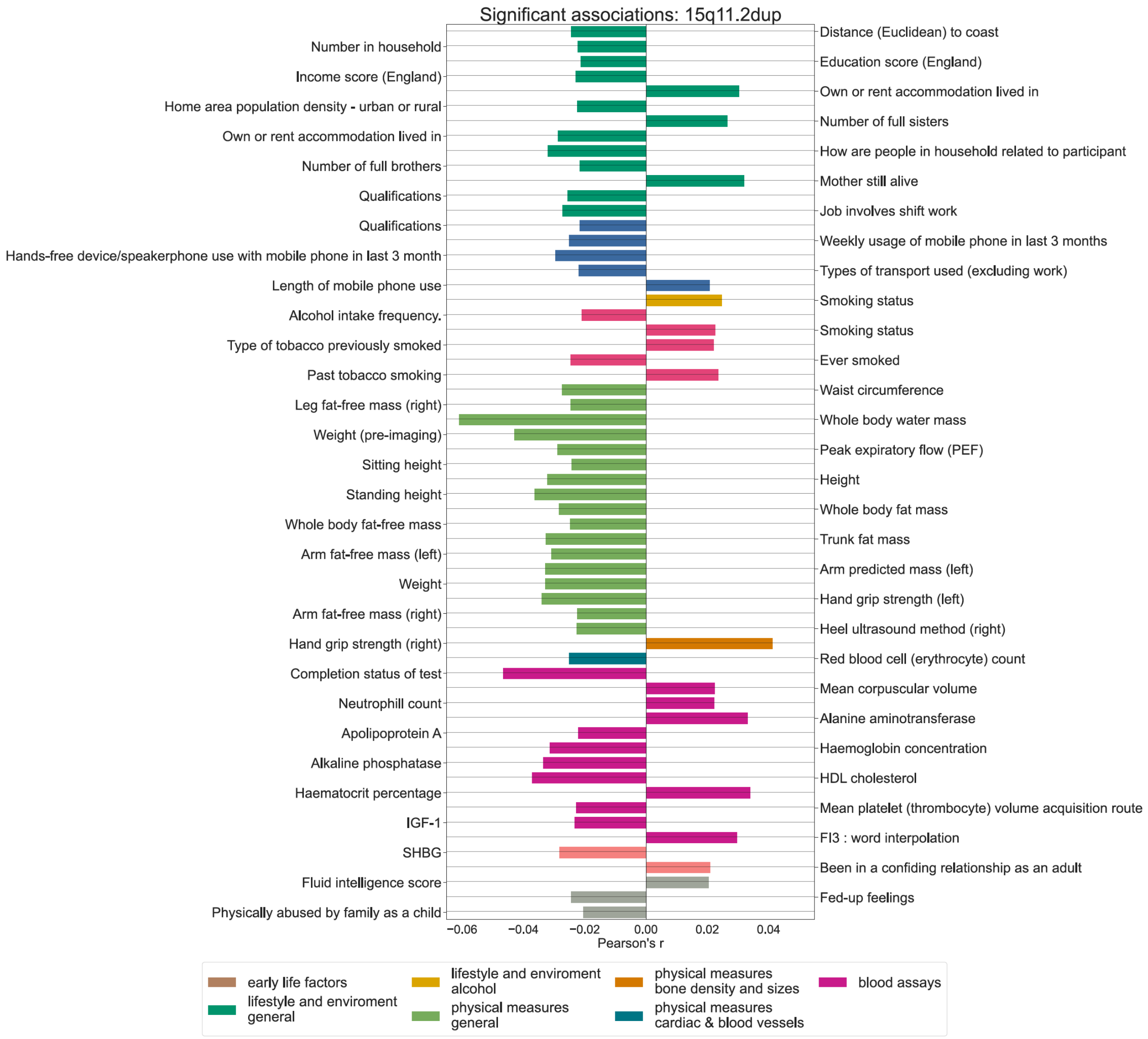

Supplementary Figure 7

Significant PheWAS associations for 15q11.2 duplication intermediate phenotype expression.

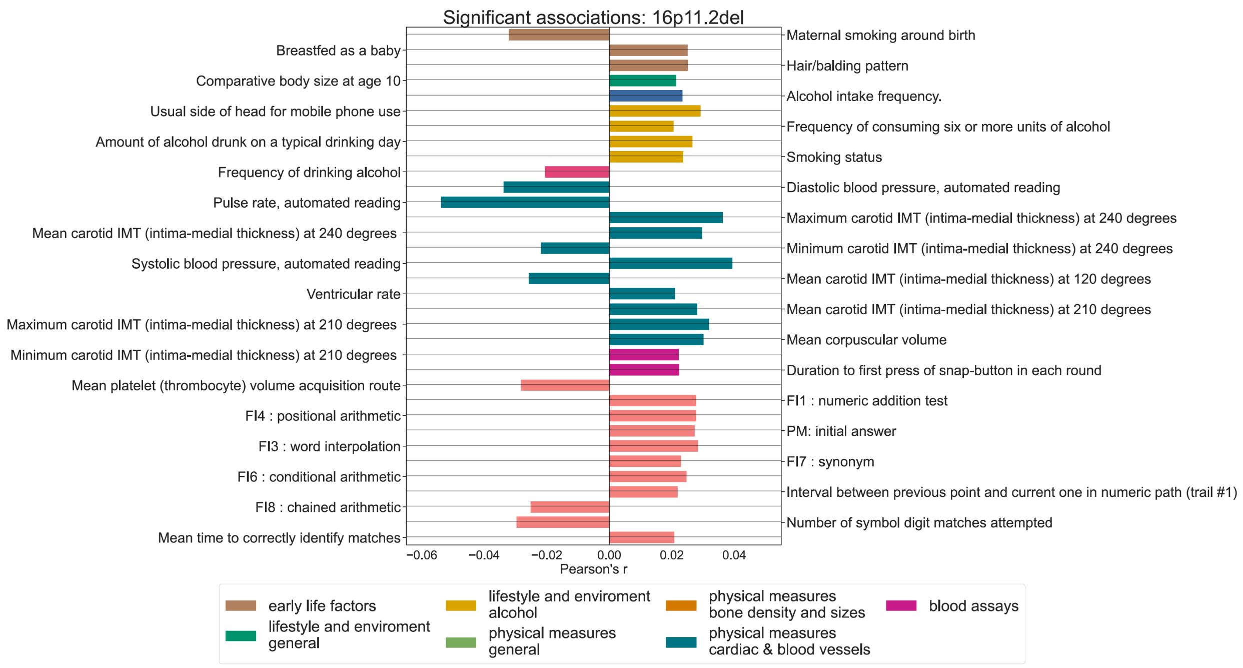

Supplementary Figure 8

Significant PheWAS associations for 16p11.2 proximal deletion intermediate phenotype expression.

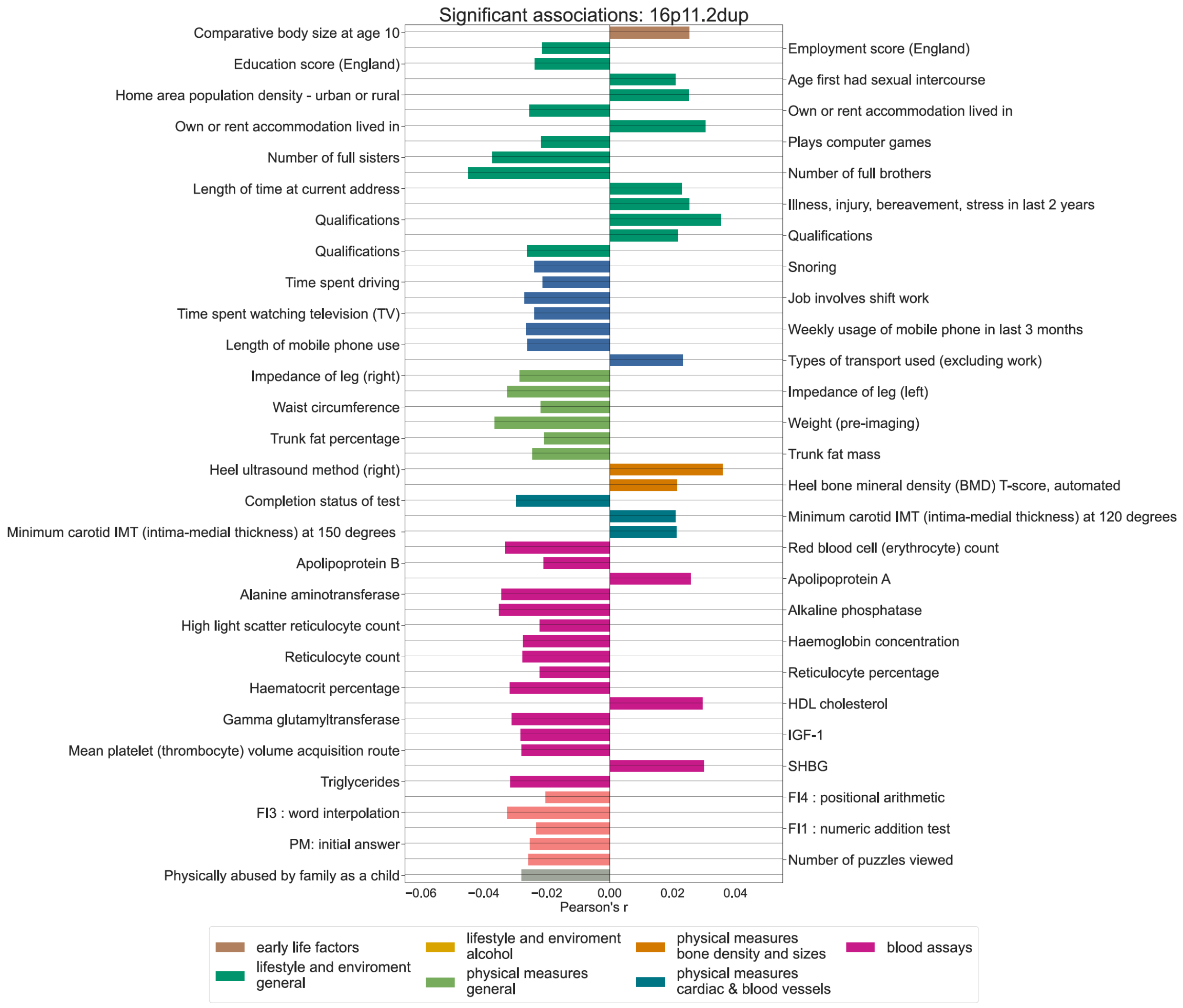

Supplementary Figure 9

Significant PheWAS associations for 16p11.2 proximal duplication intermediate phenotype expression.

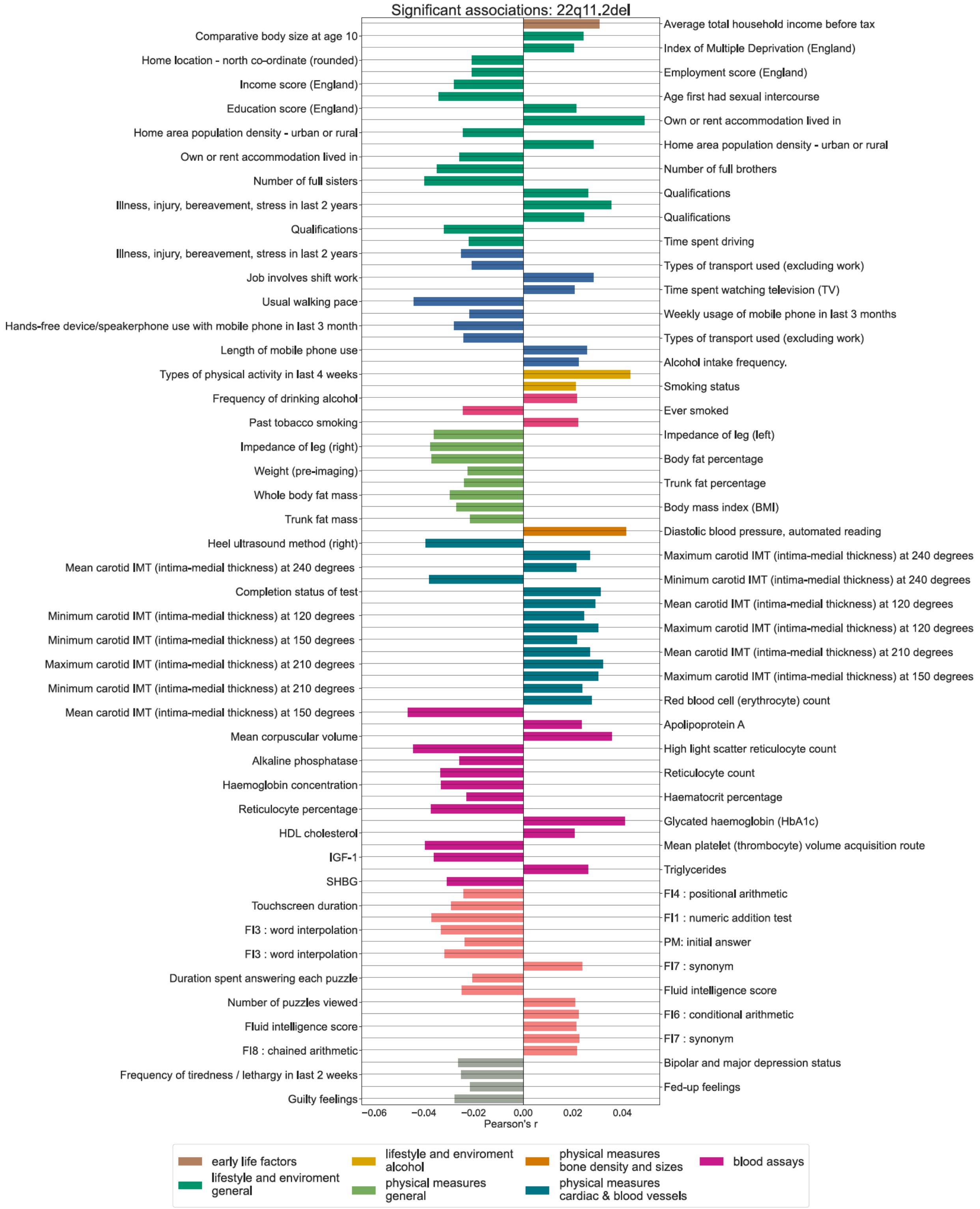

Supplementary Figure 10

Significant PheWAS associations for 22q11.2 deletion intermediate phenotype expression.

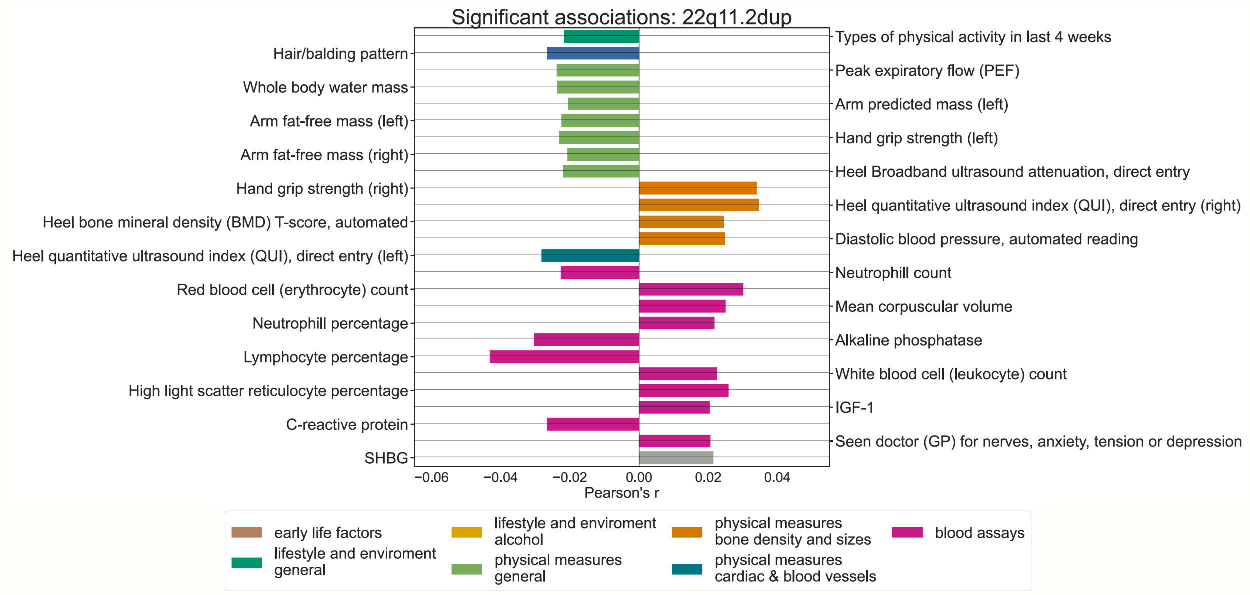

Supplementary Figure 11

Significant PheWAS associations for 22q11.2 duplication intermediate phenotype expression.

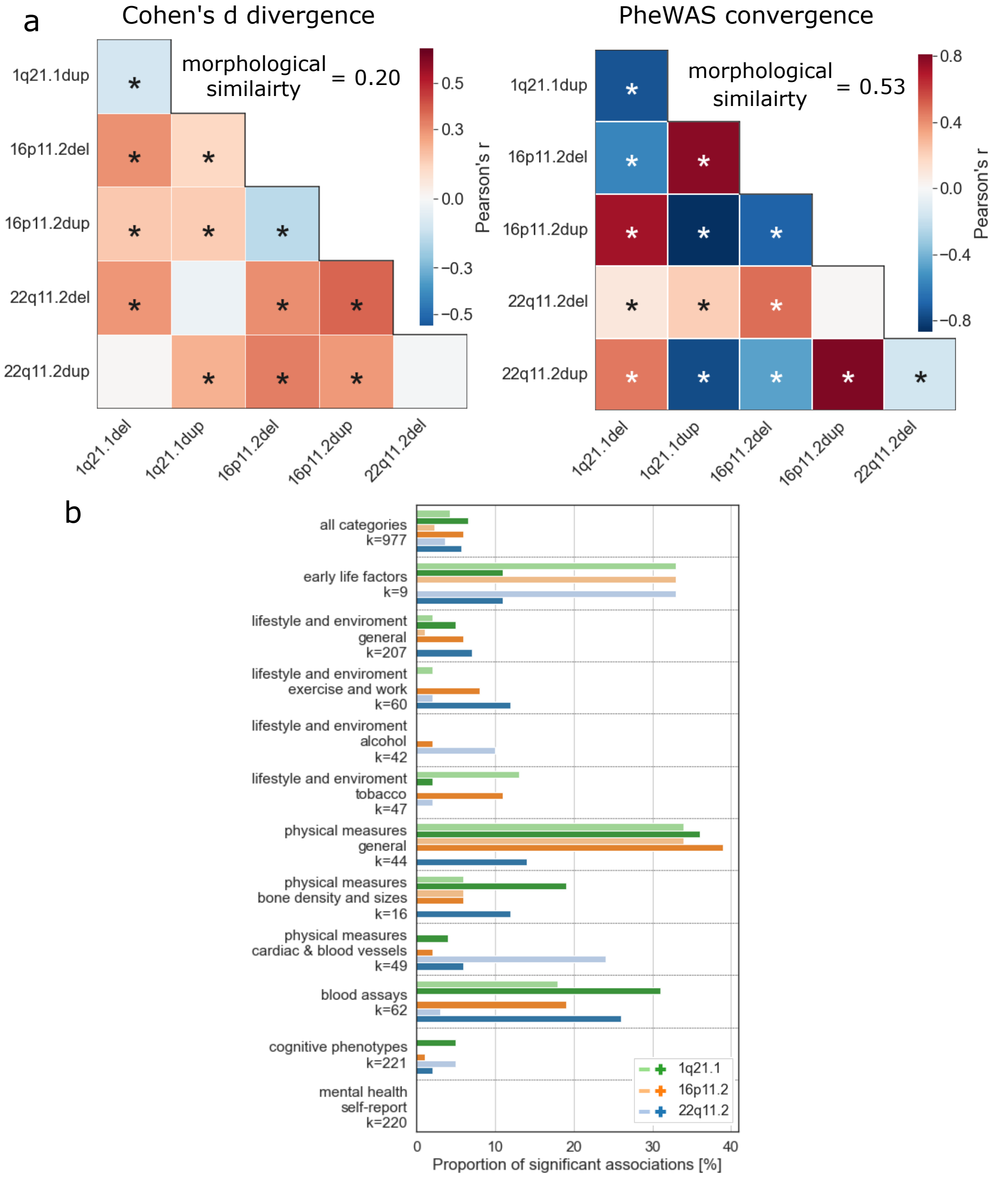

<u>Supplementary Figure 12</u>

The influence of inclusion 15q11.2 deletions and duplications from the UK Biobank.

We performed a sensitivity analysis aimed at the consequences of excluding 15q11.2 carriers from the UK Biobank and treating them as part of our clinical dataset. a) Comparison of CNV-specific Cohen's d brain maps using Pearson's correlation. Even after excluding this CNV locus from the analysis, we still observe higher phenotypic similarity compared to morphological similarity. Asterisk denotes FDR-corrected spin permutation p-values. b) Similarly, we still observe a high number of associations in physical, blood assays, or early life factors categories. The inclusion of 15q11.2 also does not affect our conclusions about the body-wide consequences of CNVs or late phenotypic convergence.

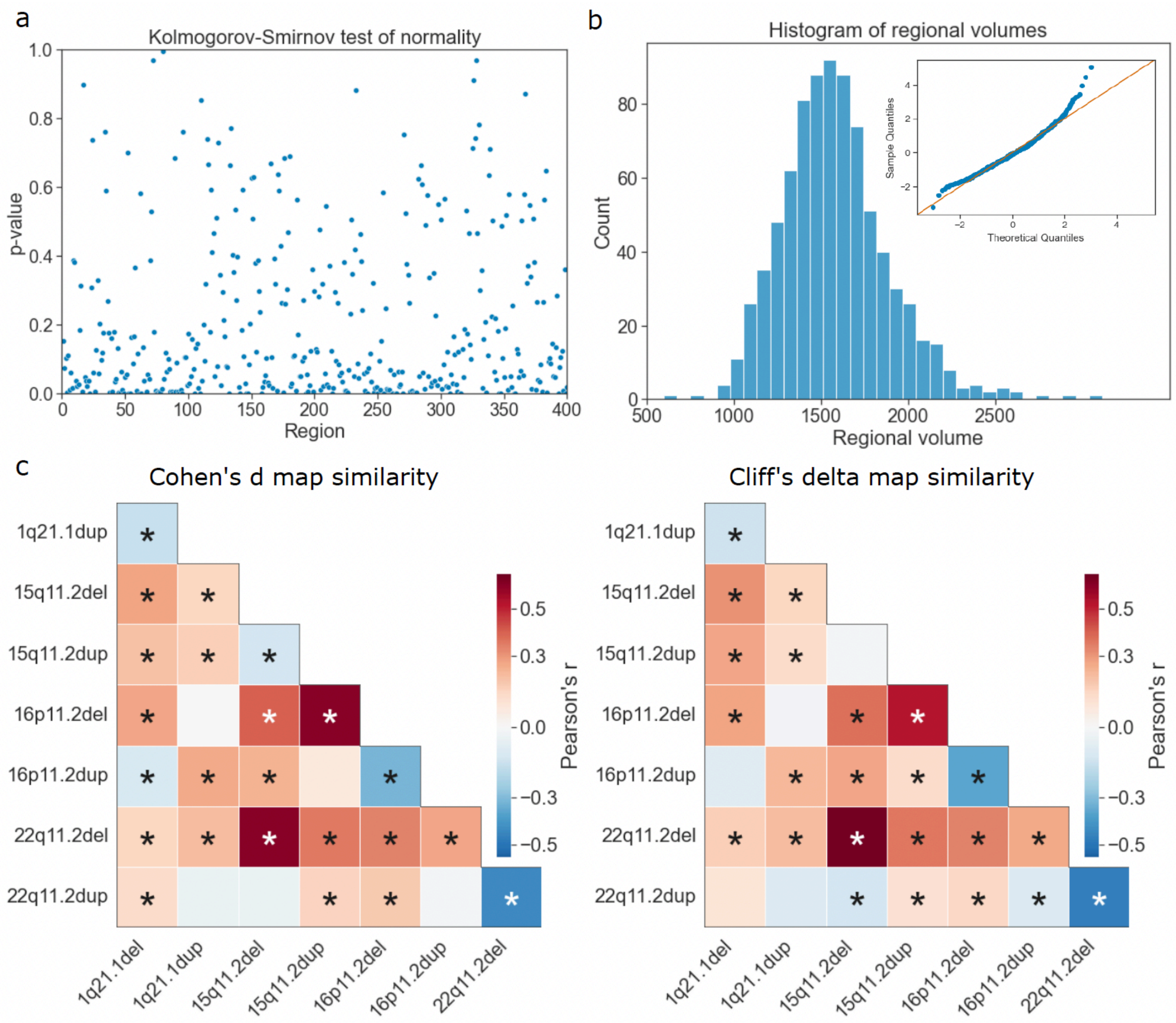

Supplementary Figure 13

Sensitivity analysis of Cohen's d results.

Replication of Cohen’s d analyses with non-parametric metric for effect size. a) Normality of regional brain volumes. The p-values of the Kolmogorov-Smirnoff goodness of fit test are presented separately for each brain region (across all subjects). The null hypothesis of a sample coming from a normal distribution cannot be rejected for most brain regions. b) Distribution of regional brain volumes across all subjects for a brain region for which we can reject the null hypothesis. The distribution still significantly resembles the Gaussian distribution, as supported by the Q-Q plot. This aspect is vital since LDA readily tolerates departures from Gaussian distribution in the individual input variables. c) Comparison of conclusions based on Cohen's d and Cliff's delta. We further replicated our results by comparing the Cohen's d brain maps from each pair of CNVs using Pearson’s correlation. The wide range and low magnitude of Pearson's correlations show that CNVs have distinct effects on brain volumes. We obtained identical results using Cliff's delta instead of Cohen's d. Again, we observe a low level of similarity between Cliff's delta brain maps and, therefore, different effects on CNVs on brain morphology. Asterisk denotes FDR-corrected spin permutation p-values.

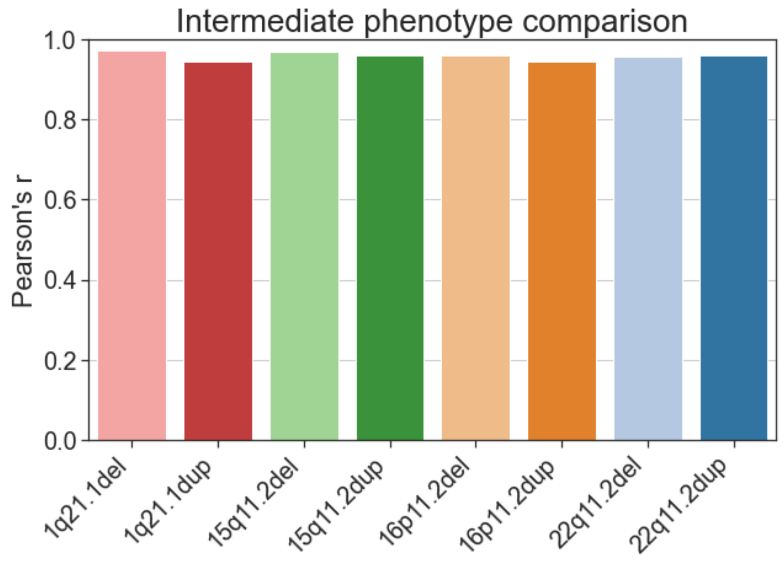

Supplementary Figure 14

Derived intermediate phenotypes independent of diagnosis.

We performed a sensitivity analysis to assess the influence of ASD and schizophrenia on derived intermediate phenotypes. We repeated the calculation of intermediate phenotypes after removing all subjects with an ASD or schizophrenia diagnosis in the clinically assessed cohort. Pearson's r corresponds to the similarity between the intermediate phenotypes from the original (Fig. 2b) and sensitivity analysis.

Consortia

16p11.2 European Consortium

Marie-Claude Addor, Joris Andrieux, Benoît Arveiler, Geneviève Baujat, Frédérique Sloan-Béna, Marco Belfiore, Dominique Bonneau, Sonia Bouquillon, Odile Boute, Alfredo Brusco, Tiffany Busa, Jean- Hubert Caberg, Dominique Campion, Vanessa Colombert, Marie-Pierre Cordier, Albert David, François-Guillaume Debray, Marie-Ange Delrue, Martine Doco-Fenzy, Ulrike Dunkhase-Heinl, Patrick Edery, Christina Fagerberg, Laurence Faivre, Francesca Forzano, David Genevieve, Marion Gérard, Daniela Giachino, Agnès Guichet, Olivier Guillin, Delphine Héron, Bertrand Isidor, Aurélia Jacquette, Sylvie Jaillard, Hubert Journel, Boris Keren, Didier Lacombe, Sébastien Lebon, Cédric Le Caignec, Marie-Pierre Lemaître, James Lespinasse, Michèle Mathieu-Dramart, Sandra Mercier, Cyril Mignot, Chantal Missirian, Florence Petit, Kristina Pilekær Sørensen, Lucile Pinson, Ghislaine Plessis, Fabienne Prieur, Alexandre Raymond, Caroline Rooryck-Thambo, Massimiliano Rossi, Damien Sanlaville, Britta Schlott Kristiansen, Caroline Schluth-Bolard, Marianne Till, Mieke Van Haelst & Lionel Van Maldergem.

Simons Searchlight Consortium

Hanalore Alupay, Benjamin Aaronson, Sean Ackerman, Katy Ankenman, Ayesha Anwar, Constance Atwell, Alexandra Bowe, Arthur L. Beaudet, Marta Benedetti, Jessica Berg, Jeffrey Berman, Leandra N. Berry, Audrey L. Bibb, Lisa Blaskey, Jonathan Brennan, Christie M. Brewton, Randy Buckner, Polina Bukshpun, Jordan Burko, Phil Cali, Bettina Cerban, Yishin Chang, Maxwell Cheong, Vivian Chow, Zili Chu, Darina Chudnovskaya, Lauren Cornew, Corby Dale, John Dell, Allison G. Dempsey, Trent Deschamps, Rachel Earl, James Edgar, Jenna Elgin, Jennifer Endre Olson, Yolanda L. Evans, Anne Findlay, Gerald D. Fischbach, Charlie Fisk, Brieana Fregeau, Bill Gaetz, Leah Gaetz, Silvia Garza, Jennifer Gerdts, Orit Glenn, Sarah E. Gobuty, Rachel Golembski, Marion Greenup, Kory Heiken, Katherine Hines, Leighton Hinkley, Frank I. Jackson, Julian Jenkins III, Rita J. Jeremy, Kelly Johnson, Stephen M. Kanne, Sudha Kessler, Sarah Y. Khan, Matthew Ku, Emily Kuschner, Anna L. Laakman, Peter Lam, Morgan W. Lasala, Hana Lee, Kevin LaGuerre, Susan Levy, Alyss Lian Cavanagh, Ashlie V. Llorens, Katherine Loftus Campe, Tracy L. Luks, Elysa J. Marco, Stephen Martin, Alastair J. Martin, Gabriela Marzano, Christina Masson, Kathleen E. McGovern, Rebecca McNally Keehn, David T. Miller, Fiona K. Miller, Timothy J. Moss, Rebecca Murray, Srikantan S. Nagarajan, Kerri P. Nowell, Julia Owen, Andrea M. Paal, Alan Packer, Patricia Z. Page, Brianna M. Paul, Alana Peters, Danica Peterson, Annapurna Poduri, Nicholas J. Pojman, Ken Porche, Monica B. Proud, Saba Qasmieh, Melissa B. Ramocki, Beau Reilly, Timothy P. L. Roberts, Dennis Shaw, Tuhin Sinha, Bethanny Smith-Packard, Anne Snow Gallagher, Vivek Swarnakar, Tony Thieu, Christina Triantafallou, Roger Vaughan, Mari Wakahiro, Arianne Wallace, Tracey Ward, Julia Wenegrat & Anne Wolken.
